# Combined Thermomechanical–Biological Treatment for Corn By-Product Valorization into Added-Value Food (Feed) Material

**DOI:** 10.3390/plants11223080

**Published:** 2022-11-14

**Authors:** Elena Bartkiene, Vytaute Starkute, Egle Zokaityte, Dovile Klupsaite, Vadims Bartkevics, Gintare Zokaityte, Darius Cernauskas, Modestas Ruzauskas, Romas Ruibys, Arturs Viksna

**Affiliations:** 1 Institute of Animal Rearing Technologies, Lithuanian University of Health Sciences, Tilzes Street 18, LT-47181 Kaunas, Lithuania; 2 Department of Food Safety and Quality, Lithuanian University of Health Sciences, Tilzes Street 18, LT-47181 Kaunas, Lithuania; 3 Institute of Food Safety, Animal Health and Environment BIOR, Lejupes iela 3, LV-1076 Riga, Latvia; 4 Food Institute, Kaunas University of Technology, Radvilenu Road 19, LT-50254 Kaunas, Lithuania; 5 Faculty of Veterinary, Institute of Microbiology and Virology, Lithuanian University of Health Sciences, Tilzes Street 58, LT-47181 Kaunas, Lithuania; 6 Institute of Agricultural and Food Sciences, Agriculture Academy, Vytautas Magnus University, K. Donelaicio Street 58, LT-44244 Kaunas, Lithuania; 7 Department of Chemistry, University of Latvia, Jelgavas Street 1, LV-1004 Riga, Latvia

**Keywords:** corn, by-products, extrusion, fermentation, safety, valorization

## Abstract

The aim of this study was to apply the combined thermomechanical–biological treatment for corn processing by-product (CPBP) valorization to added-value food and feed material. The mechanical–thermal pre-treatment was performed by applying the extrusion technique. Extruded CPBPs (14, 16, and 18% moisture) were further biodegraded with *Lactiplantibacillus plantarum-LUHS122* (Lpl), *Liquorilactobacillus uvarum-LUHS245* (Lu), *Lacticaseibacillus casei-LUHS210* (Lc), and *Lacticaseibacillus paracasei-LUHS244* (Lpa). Acidity parameters, microbial characteristics, sugars concentration, amino and fatty acids profile, biogenic amines (BA), and antibacterial and antifungal properties of CPBP were analyzed. Fermented CPBP had a reduced count of mould/yeast. A significantly lower (*p* ≤ 0.05) count of total enterobacteria was found in most of the extruded–fermented CPBP. Fermentation of extruded CPBP (moisture of 16 and 18%) increased valine and methionine content. Cadaverine and spermidine were not found after treatment of CPBP, and the lowest content of BA was found in the extruded–fermented (Lpa, moisture 18%) CPBP. Applied treatment had a significant effect on most of the fatty acids. CPBP fermented with Lpl, Lu, and Lpa displayed inhibition properties against 3 of the 10 tested pathogenic/opportunistic bacterial strains. Extruded–fermented (Lu, Lc, and Lpa moisture of 14 and 18%) CPBP showed antifungal activity against *Rhizopus.* Extruded–fermented (14% moisture, Lpl) CPBP inhibited *Rhizopus* and *Aspergillus fumigatus*. In conclusion, combined treatment can improve certain parameters and properties of CPBP in order to produce safer and more nutritious ingredients for food and feed industries.

## 1. Introduction

Cereal grains are staple crops and provide food and energy for the population year-round because they are easy to store, and maintain essential nutrients for humans and animals [1,2]. The most important part of the cereal is starchy endosperm; however, most of the functional compounds are generally located in the outer part of the grain. Despite that, the utilization of cereal grain outer part in the food industry is very low (on average, 7.5%) due to the negative effects on overall acceptability of the product [1]. In addition to wheat, rye, and rice, corn (*Zea mays* L.) is cultivated globally in many regions [3,4]. Corn grain outer layer matrix is very complex, and contains hemicellulose, cellulose, protein, starch, crude oil, and phenolic acids [5]. Various by-products are obtained after corn starch processing (e.g., corn bran, steep liquor, corn germ, gluten meal, etc.) and most of them are used as livestock or poultry feed or valorized into valuable products for food and medicine industry, such as edible oils, dietary fibre, sterol, ferulic acid, proteins, zeaxanthin, and active polysaccharides [6,7,8]. Chemical composition of the corn by-products is also suitable for biological treatment, because all the composites are good energy sources for microorganism biomass cultivation in the fermentation industry. Only 18% of corn production is used for human nutrition, while the rest is dedicated to animal feed [9]. However, some part of the non-starch by-products are still not fully utilized [10]. Moreover, the important point is the efficiency of the valorization of these by-products while maintaining the quality of the final product. Despite that some valorization technologies are developed and used for higher value product preparation and sustainable processing, which is based on economic efficiency and environmentally friendly production, is still challenging. Some methods for the preparation of high value-added products include extraction, and biorafination steps, which are not economically efficient, and could not be easily adapted at an industrial scale.

Extrusion is applied in the food industry due to low cost, high production rate, and energy efficiency [11]. Extrusion induces physicochemical changes in processed material, e.g., starch gelatinization, denaturation of proteins, amylose–lipid complex formation, inactivation of enzymes and microorganisms [12]. Studies on the extrusion of various corn products (flour, meal, grits, starch, and gluten meal) and their combination with other materials such as brewer’s spent grain, sugar beet pulp, apple pomace, sweet potato, and soybean flour, have been conducted [12]. Fermentation with lactic acid bacteria (LAB) is another promising technique, which leads to decreases in the level of fermentable carbohydrates, increases in total soluble solids, free amino acids, etc., functional compounds in fermented substrate, and can be used as a single treatment or in combination with other techniques [13]. Taking into consideration that microbial contamination of by-products could be a problem to ensure domination of the technological microorganisms in fermentable substrate, extrusion pre-treatment can be a valuable step to decontaminate them in order to ensure stability of the process. As well as extrusion, fermentation could improve the digestibility of protein and starch, and this would be beneficial for the production of low-cost, nutritionally enriched food and feed ingredients [1,14]. However, little data are available in the literature on the changes in such corn by-products as bran and germ after fermentation. To the best of our knowledge, there is no data about the combined technique of extrusion and fermentation for these by-products processing.

Therefore, our hypothesis is that the valorization of corn processing by-products could be designed in a more appropriate and sustainable manner by using whole by-product conversion, by combining extrusion and fermentation processes, as the latter are common and economically efficient processes in the food and feed industry. The appropriate selection of the technological microorganisms, which possess antimicrobial properties, for corn by-product fermentation could lead to the production of a functional material with additional desirable antibacterial and antifungal properties for the food and feed industry. In this study, combined thermomechanical–biological treatment for corn cereal grain processing by-product valorization to added-value food and feed material was tested. Extruded corn by-products with moisture content of 18, 16, and 14% were biodegraded with antimicrobial properties possessing *Lactiplantibacillus plantarum-LUHS122*, *Liquorilactobacillus uvarum-LUHS245*, *Lacticaseibacillus casei-LUHS210*, and *Lacticaseibacillus paracasei-LUHS244*. To select the most appropriate technique for corn by-product valorization, acidity parameters (pH, total titratable acidity, lactic acid concentration), microbial characteristics (LAB, mould/yeast, total bacteria count, total enterobacteria count), sugars concentration (fructose, glucose, sucrose, maltose), amino and fatty acids profile, biogenic amines concentration, and antibacterial and antifungal properties of the prepared samples were analyzed.

## 2. Materials and Methods

### 2.1. Corn By-Products and Technological Microorganisms Used in Experiments

**Figure 1:**
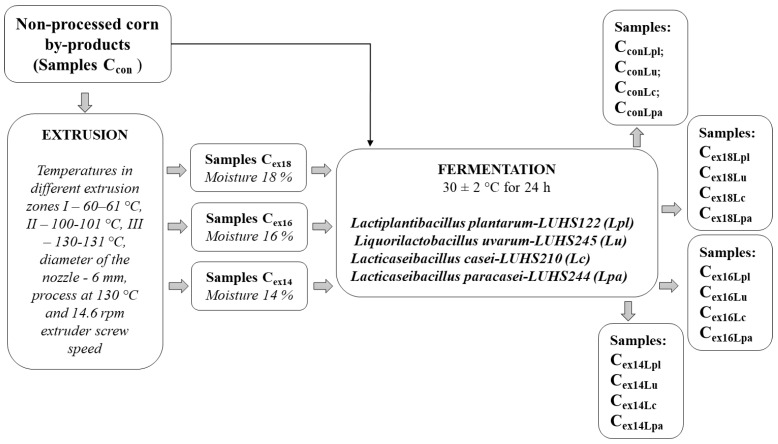
The principal scheme for corn by-products valorization.

The principal scheme for corn by-products valorization is given in Figure 1.

Corn by-products, non-processed, and extruded in a Twin Screw extruder (Jinan Shengrun Machinery Co., Ltd., Jinan, China), were obtained from SME “Ustukiu malunas” (Pasvalys, Lithuania). The temperatures in the different extrusion zones were I—60–61 °C, II—100–101 °C, and III—130–131 °C. Different moisture contents of the corn by-products substrate during the extrusion were tested (18, 16, and 14%). Extruder feed rate (F) was 8.2 ± 0.3 kg/h, and the nozzle diameter was 6 mm. The moisture content of the final corn by-product samples (after extrusion) was 11%. The samples were extruded at 130 °C and 14.6 rpm extruder screw speed. Three extruded corn by-product sample groups were prepared (C_ex18_, C_ex16_, C_ex14_) and non-extruded corn by-product samples were used as a control (C_Con_).

The LAB strains *Lactiplantibacillus plantarum*-LUHS122, *Liquorilactobacillus uvarum*-LUHS245, *Lacticaseibacillus casei*-LUHS210, and *Lacticaseibacillus paracasei*-LUHS244 were used for the fermentation of C_Con_, C_ex18_, C_ex16_, C_ex14_. Characteristics, including carbohydrates metabolism, survival at low pH, gas production capacities, and antimicrobial and antifungal properties, of the LAB strains, used for corn by-product fermentation, are reported by Bartkiene et al. [15]. Prior to the experiments, LAB strains were multiplied in MRS broth (de Man–Rogosa–Sharpe, CM 0359, Oxoid Ltd., Hampshire, UK) at 30 ± 2 °C for 48 h. The corn by-products, water, and a suspension of LAB strain (3% of dry matter relative to the corn by-product mass) containing 8.9 log10 CFU/mL were incubated at 30 ± 2 °C for 24 h. For 100 g of corn by-product, 60 mL of water was used. Three parallel replicates of the fermentation were performed, and three parallel samples were analyzed.

### 2.2. Analysis of the Acidity Parameters and Microbiological Characteristics

The pH was measured using a pH electrode (PP-15; Sartorius, Goettingen, Germany). The total titratable acidity (TTA) was evaluated for a 10 g portion of sample mixed with 90 mL of water; the results were expressed as mL of 0.1 mol/L NaOH solution required to achieve a pH value of 8.2. The concentration of L-(+) and D-(−)-lactic acid isomers was evaluated using a specific Megazyme assay Kit (Megazyme, Bray, Ireland). LAB, total bacteria (TBC), enterobacteria (TEC), and mould/yeast (M/Y) counts in the samples were determined according to Bartkiene [16].

### 2.3. Analysis of the Amino Acids Profile and Biogenic Amines Concentration

For amino acid analysis, analytes were extracted from homogenized sample with aqueous 0.1 M HCl solution and dansylation were performed according to the method of Hua-Lin Cai et al. [17], with some modifications. The concentrations of analytes were determined using The Varian ProStar HPLC system (Varian Corp., Palo Alto, CA, USA) and Thermo Scientific LCQ Fleet Ion trap mass detector. The detailed description of the method is given in Supplementary Data S1.

Biogenic amines (BA) were analyzed according to the method of Ben-Gigirey et al. [18] with some modifications by Bartkiene et al. [19]. Following BAs were analyzed: tryptamine, phenylethylamine, cadaverine, putrescine, histamine, tyramine, spermine (SPER), and spermidine. The extraction of BA was performed by using 0.4 M perchloric acid. The derivatization was carried out with a dansyl chloride solution in acetonitrile (10 mg/mL). The content of each BA was analyzed with the Varian ProStar HPLC system (Varian Corp., Palo Alto, CA, USA). The detailed description of the method is given in Supplementary Data S1.

### 2.4. Determination of Sugars Concentration in Corn By-Product Samples

Sugars concentration analysis of the non-treated and treated corn by-products was carried out with an Ultra Performance Liquid Chromatography system (Shimadzu Corp., Kyoto, Japan). A 2 mg/mL standard solution of a sugar mixture (fructose, glucose, sucrose, and maltose) was used for sugar detection. The detailed description of the method is given in Supplementary Data S1.

### 2.5. Evaluation of Fatty Acids Profile

The fatty acid (FA) composition of the corn by-product samples was determined using GCMS-QP2010 (Shimadzu, Japan) gas chromatograph with a mass spectrometer. The FA methyl esters (FAME) concentration was determined using 3-point calibration curve method and results were expressed as the percentage of total FAME concentration in the sample. The detailed sample preparation and chromatographic conditions is given in Supplementary Data S1 and Supplementary Data S3.

### 2.6. Evaluation of Antimicrobial Properties

The antibacterial activity of the non-treated and treated corn by-products against a variety of pathogenic and opportunistic bacterial strains was assessed by measuring the diameter of inhibition zones (DIZ, mm) in agar well diffusion assays. The list of pathogenic and opportunistic bacterial strains and detailed description of the method is given in Supplementary Data S1.

The antifungal activities of the non-treated and treated corn by-products against 10 different mould species were determined by the agar well diffusion assay [20]. The list of mould species and detailed description of the method is given in Supplementary Data S1.

### 2.7. Statistical Analysis

The physico-chemical data were expressed as the mean values (*n* = 3) of each sample ± standard error (SE), and the microbiological data were expressed as the mean values (*n* = 5) of each sample ± standard error (SE). The effects of the different treatments were analyzed by multivariate analysis of variance (ANOVA) and Tukey’s honestly significant difference test (HSD) procedure, as post-hoc tests. A linear Pearson’s correlation was used to quantify the strength of relationships between variables. The correlation coefficients were calculated using the statistical package SPSS for Windows (v15.0, SPSS, Chicago, IL, USA). Correlation strength interpretation was performed in accordance with Evans et al. [21]. The results were recognized as statistically significant at *p* ≤ 0.05.

## 3. Results and Discussion

### 3.1. Acidity Parameters and Microbiological Characteristics of the Corn By-Products

The changes in acidity parameters of corn by-product after fermentation are shown in Table 1. After 24 h of fermentation, the significant reduction in pH values and increase in TTA values of all samples were observed, compared to non-fermented samples. The lowest pH after 24 h of fermentation was found in the C_conLpl_, C_conLu_, and C_conLc_ samples (3.34, 3.35, and 3.30, respectively) and the highest TTA was found for C_ex18Lpl_ (5.5° N).

The content of L(+)- and D (−)-lactic acid isomers after 24 h of fermentation varied from 0.274 to 0.360 and 0.321 to 0.6 g/100 g, respectively. The highest content of L (+) isomer was produced in C_conLpa_ and C_ex14Lpa_ (0.360 and 0.362 g/100 g, respectively). The lowest content of D (−) isomer was found in C_ex18Lpa_ (0.056 g/100 g).

The results also showed that increased moisture content of extruded corn by-products increased the pH and decreased TTA of samples, as well as the concentration of lactic acid isomers. At 16 and 18% corn by-products moisture, the pH of the fermented samples was the highest, and the TTA was the lowest compared to corn extrudates with lower moisture content (Table 1). The concentration of lactic acid isomers was lower at higher moisture (18%) content of corn extrudates and varied from 0.095 to 0.221 g/100 g and 0.056 to 0.251 g/100 g, L (+) and D (−) content, respectively, compared to corn extrudates with moisture of 14% (Table 1).

Grain seeds fermentation is related with increased nutritional value, a high number of viable LAB, reduced pH, and a high concentration of organic acids [22,23,24]. In a previous study, the increase in TTA values with increasing fermentation time supported the decrease in pH performance, which was one of the most important changes during LAB fermentation [25,26]. Fast acidification by starter cultures, resulting in pH reduction, is considered critical from a food safety standpoint and plays a vital role in eliminating food pathogens and extending product quality [27,28].

Microbiological parameters of non-treated and treated corn by-products are shown in Table 2. Lactic acid bacteria (LAB) counts were significantly higher in the fermented (non-extruded) and extruded–fermented corn by-products, compared to the control samples. The highest LAB count was found in C_conLc_ (9.39 log10 CFU/g). LAB count in the extruded (non-fermented) samples was lower or similar to that of the control samples. In corn by-products fermented with Lpl, Lu, and Lc, mould/yeast (M/Y) counts (3.72–3.80 log10 CFU/g) were significantly lower compared to the control. M/Y was significantly higher in most of the other extruded and extruded–fermented samples, compared to control. Significantly lower total bacteria count (TBC) was found in most of the fermented (C_conLpl_, C_conLu,_ and C_conLpa_) and extruded–fermented (C_ex14Lpl_, C_ex14Lu_, C_ex16Lpa_, C_ex18Lu_, C_ex18Lc_, and C_ex18Lpa_) samples, compared to the control group, with the lowest (7.94–8.12 log10 CFU/g) being in C_conLu_, C_ex18Lu_, and C_ex18Lpa_. Total enterobacteria count (TEC) was significantly lower in all the fermented (non-extruded) samples and most of the extruded–fermented samples (C_ex14Lc_, C_ex14Lu_, C_ex16Lpl_, C_ex16Lu_, C_ex16Lc_, C_ex18Lpl_, C_ex18Lu_, C_ex18Lc_, and C_ex18Lpa_), with the lowest being in C_ex16Lc_ and C_ex18Lpl_ (5.45 and 5.32 log10 CFU/g). The extruded (non-fermented) samples had significantly higher TEC than control.

The *Lactobacillus* group produces many antimicrobial compounds, including lactic and acetic acids, that reduce environmental pH and are antagonistic to a wide range of pathogenic and opportunistic microorganisms [29]. Organic acids, produced by LAB, lower environment pH, and limit the growth of bacterial pathogens [30]. Our results are similar with Ayyash et al. [31], who found that compared with day 0, all *Lactobacillus* spp. populations increased (*p* < 0.05) in all grain ferments. In general, *Lactobacillus* spp. increased by ~1.5 log (~7.0 logs to 9.0 logs) during 48 days of storage. The similar results were observed by Ferrero et al. [32], who reported that the yeast count decreased with fermentation and was below the detection limit at 250 days, while the mould count was under the detection limit after 30 days of fermentation, regardless of the treatment.

### 3.2. Amino Acids Profile and Biogenic Amines Formation in Corn By-Products

Essential amino acids (EAA) mass concentrations in corn by-products are presented in Table 3. The predominant EAA in control group were isoleucine (Ile), valine (Val), tryptophan (Trp), and threonine (Thr) and their content ranged from 0.15 to 0.36 g/100 g. Lysine was not found in all samples, while the presence of leucine (<0.02 g/100 g) was observed in all fermented (not extruded) samples, as well as extruded–fermented C_ex14Lpl_ and C_ex14Lu_ samples. Significant changes (*p* ≤ 0.05) were found in the contents of all EAA between the tested samples. Extrusion increased the content of Phe and Val in C_ex14_ and C_ex18_, respectively, as well as Met in C_ex16_ and C_ex18_, compared to the control samples. Fermentation of the control samples increased the contents of Phe and histamine (His). Fermentation of the extruded samples increased the contents of Phe, His, and Thr in C_ex14Lpl_; Val in C_ex14Lpl_ and all extruded samples with moisture content of 16 and 18%; methionine in all extruded samples with moisture content of 16 and 18%, compared to control. The contents of Trp and Ile were reduced or similar in fermented, extruded, or extruded–fermented samples, compared to control.

Non-essential amino acid (NEAA) concentrations in corn by-products are given in Table 4. The presence of arginine (<0.08 g/100 g) was observed in all fermented (not extruded) samples, as well as extruded–fermented C_ex14Lpl_, C_ex14Lu_, and C_ex16Lc_ samples. The contents of alanine and proline were reduced in the fermented, extruded, or extruded–fermented samples, compared to the control. Tyrosine (Tyr) and glutamine (Glu) were reduced in all the extruded and extruded–fermented samples, compared to the control. However, fermented (non-extruded) corn by-products shared similar contents of Tyr and Glu with the control group. After treatment of corn by-products, arginine appeared in all fermented (non-extruded) samples, as well as in some extruded–fermented samples (C_ex14Lpl_, C_ex14Lu_, and C_ex16Lc_). Aspartic acid and glycine were significantly higher in fermented (non-extruded) samples and C_ex14Lpl_, compared to the control group. Serine was significantly higher in C_conLu_ and C_ex14Lpl_, compared to the control group. Cysteine (Cys) was significantly higher in all extruded and extruded–fermented samples with moisture content of 16 and 18%, compared to the control group. The highest content (1.40–1.69 g/100 g) of Cys was found in the extruded–fermented corn by-products with a moisture content of 18%.

The increased content of some amino acid in fermented corn by-products could be explained by the proteolytic activity of LAB and endogenous proteases which are activated under the acidic conditions [33]. Moreover, certain amino acids and peptides are also used by LAB for their metabolism. Our results partly agree with Onyango et al. [34], that aspartic acid, glycine, cystine, and methionine increased after fermentation, while contents of all other amino acids showed no significant changes. Another study showed that fermentation of corn milling by-products improved the content of free amino acids and polypeptides [35]. The increased content of free amino acids after wheat bran fermentation with LAB was also observed [36]. Protein structure could be destroyed due to the conditions of the extrusion process as high temperature and pressure [37]. Thermal degradation of lysine, valine, leucine, threonine, and isoleucine was reported [14]. It was found that the extrusion of wheat bran led to a quantitative decrease in amino acids and the protein and moisture content of raw material, while the barrel temperature had no significant influence on cysteine and methionine content in rice-based snacks [38,39]. Moisture levels also influence lysine retention, but conflicting results have been observed [40]. However, the increase in the concentration of amino acids (for arginine, histidine, proline, and alanine by 80, 3, 13, and 11%, respectively) in corn after extrusion, relative to the native form, was observed by Kholodilina et al. [38]. It was also reported that extrusion increased all amino acids, except Lys and Pro in corn [41].

**Figure 2:**
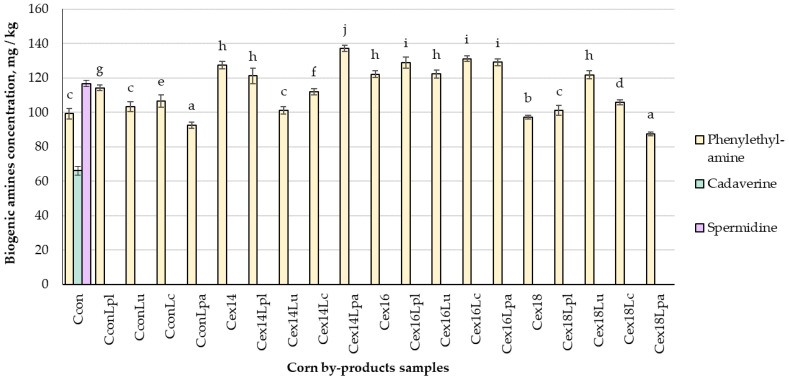
Biogenic amines mass concentration in corn by-products C—corn by-product samples; con—control samples (non-extruded non-fermented); Lpl, Lu, Lc, Lpa—fermented with *L. plantarum*-LUHS122, *L. uvarum*-LUHS245, *L. casei*-LUHS210, *and L. paracasei*-LUHS244 strains, respectively; ex—extruded samples; 14, 16, 18—moisture content of the corn by-product samples. Data are represented as means (*n* = 3) ± SE. a–j—mean values denoted with different letters are significantly different between samples (*p* ≤ 0.05).

The concentrations of BAs in corn by-products that were extruded and fermented by LAB, are presented in Figure 2. Histamine, tryptamine, putrescine, tyramine, and spermine were not found in all the tested samples. Phenylethylamine (PHE) was found in all corn by-products, while cadaverine and spermidine were found only in non-treated samples. Results showed that treatment had a significant effect (*p* ≤ 0.05) on the concentration of PHE in corn by-products. Fermentation with Lpl and Lc strains, as well as extrusion (moisture content of 14 and 16%) of control samples significantly increased PHE content by on average 18.4%. However, after extrusion, PHE was significantly lower in the C_ex18_ sample, compared to the control group. In most of cases, fermentation of the extruded samples led to the increased content of PHE, compared to only extruded corn by-products or control group. Fermentation with *L. paracasei*-LUHS244 significantly reduced the content of PHE in C_conLpa_ and C_ex18Lpa_ samples, compared to non-treated corn by-products and that was the lowest concentration of BA found between all samples.

Biogenic amines (BA) are low-molecular-weight nitrogenous organic bases, which can accumulate in high concentration in food due to microbial activity and cause toxic effects in consumers. Some food microorganisms are able to degrade BA once they have been synthesized in the food matrix [42,43]. Corn can also be a source of biogenic amines. Some biogenic amines can be naturally present in corn whereas others can be introduced during production, processing, and storage. They can be formed by thermal or microbial decarboxylation of amino acids and may be used as an index of quality or hygienic conditions of products [44]. Cadaverine and spermidine can be naturally found in raw plant foods, while PHE can be produced by microorganisms from phenylalanine [45,46].

The reports on the effect of extrusion processing or LAB fermentation on biogenic amine changes in corn products are scarce. Previous research reported that no significant changes in the formation of amines occurred during brewing fermentation, except for tryptamine and tyramine [47]. In the another study, the content of PHE in hemp (*Cannabis sativa* L.) seed paste fermented with *L. uvarum*-LUHS245 and *L. casei*-LUHS210 increased by 5% and decreased by 52%, respectively, compared with untreated samples [48].

### 3.3. Sugars Concentration in Corn By-Product Samples

**Figure 3:**
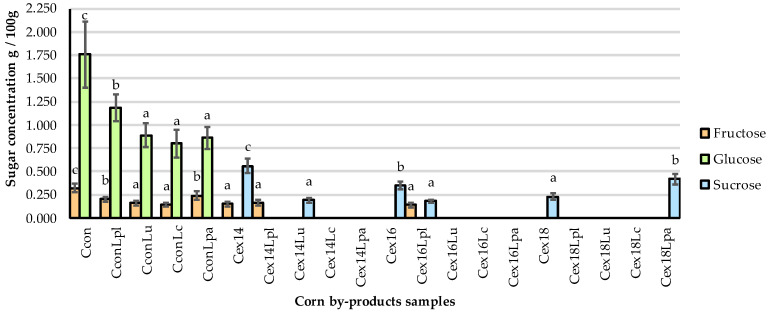
The sugars mass concentration in corn by-products. C—corn by-product samples; con—control samples (non-extruded, non-fermented); Lpl, Lu, Lc, Lpa—fermented with *L. plantarum*-LUHS122, *L. uvarum*-LUHS245, *L. casei*-LUHS210, and *L. paracasei*-LUHS244 strains, respectively; ex—extruded samples; 14, 16, 18—moisture content of the corn by-product samples. Data are represented as means (*n* = 3) ± SE. a–c—mean values denoted with different letters are significantly different between samples (*p* ≤ 0.05).

The contents of fructose, glucose, sucrose, and maltose in non-treated and treated corn by-products were analyzed and the results are given in Figure 3. There was a significant effect (*p* ≤ 0.05) of treatment on the fructose, glucose, and sucrose concentration in corn by-product samples. Significantly lower (*p* ≤ 0.05) contents of fructose and glucose (on average by 42.2 and 47%, respectively) were found in all fermented (non-extruded) corn by-products, compared to non-treated samples. The same tendency was also observed with fructose content in C_ex14_, C_ex14Lpl_, and C_ex16Lpl_ samples (lower, on average, by 53.1%), while fructose was not determined in the rest of samples. Glucose was not found in all extruded and extruded–fermented samples. The presence of sucrose was observed after extrusion in C_ex14_, C_ex16_, and C_ex18_. After fermentation of the extruded samples, the content of this sugar was found to be significantly lower (*p* ≤ 0.05) in C_ex14Lu_ and C_ex16Lpl_ by 66.1 and 48.6%, respectively, compared to only extruded samples. Maltose was not found in any of the tested samples.

The variations of fructose, glucose, and sucrose concentrations in fermented corn by-products are related with the LAB activity. During fermentation, the conversion of available carbohydrates by LAB, which could also possess polysaccharide-degrading activity, yields such compounds as lactic, and acetic acid [49,50]. As carbohydrates undergo a series of changes under the extrusion conditions, the lower concentrations of fructose, the absence of glucose and a higher content of sucrose in extruded corn by-products could be explained by the influence of extrusion processes. Due to the impact of shear forces, as well as feed moisture content and temperature, the insoluble fibres in corn by-products could be broken down into soluble compounds with reduced molecular weight [51]. Lower moisture content in feed causes a more intensive decomposition of insoluble dietary fibres [11]. Moreover, the formation of Maillard browning products during extrusion lowers the content of reducing sugars in corn by-products.

### 3.4. Corn By-Products Fatty Acids Profile

The fatty acids (FA) profile of non-treated and treated corn by-products is given in Table 5 and Supplementary Data S2. The FA profile of samples revealed that linoleic acid (C18:2 n6, 41–53%) was the highest followed by oleic (C18:1 n9, 32–33%), palmitic (C16:0, 9–13%), stearic (C18:0, 2–4%), and α-linolenic (C18:3 n3, 1–3%) acids (Supplementary Data S2). Similar results have been reported for predominant FA by other authors [52,53]. The rest of FA were observed at low levels (less than 1%). It was found that treatment had a significant effect (*p* ≤ 0.05) on most of the FA (C4:0, C6:0, C8:0, C10:0, C12:0, C14:0, C15:0, C15:1, C16:0, C16:1, C17:0, C18:0, C18:3, C20:1, C21:0, C20:2, C22:0, C20:3, C24:0, and C22:6) in corn by-products. Palmitic and stearic acids were significantly higher in C_ex18Lpl_ and C_ex18Lc_, compared to the rest of the samples. Compared to non-treated corn-by products, α-linolenic was significantly higher in extruded and extruded–fermented samples, with C_ex18Lu_, C_ex18Lc_, and C_ex18Lpa_ being the highest. In the case of other FA, clearer tendencies cannot be drawn. However, significant differences in the content of oleic and linoleic acid between samples were not found.

The FA profile of all corn by-products was dominated by polyunsaturated FA (PFA, 45–54%), followed by monounsaturated FA (MFA, 33–35%), and then saturated FA (SFA, 13–21%) (Table 5). Significantly higher content of SFA was found in several extruded–fermented C_ex14Lc_, C_ex18Lpl_ C_ex18Lc_, compared to the rest of the samples (*p* ≤ 0.05), while MFA content was similar in all tested corn by-products. PFA content was significantly lower (*p* ≤ 0.05) only in C_ex16Lu_, compared to other samples. The higher content of PFA is favourable in food because they improve blood sugar level, possess blood cholesterol and pressure lowering abilities, and fight against inflammatory reactions and various cancers [54]. The PFA/SFA ratio ranged from 2.2 to 4.2 with the highest being for C_conLpl_, C_conLpa_, and C_ex14Lpl_ and the lowest being for C_ex18Lpl_ and C_ex18Lc_. Ratios of all tested samples were higher than 0.4 as recommended by the World Health Organization [55]. The group of omega 6 FA was the highest (41–53%) followed by omega 9 (32–34%), and omega 3 (1–4%) in all tested samples (Table 5). Significant changes in the content of omega 6 and omega 9 were not observed in all tested samples. However, compared to non-treated samples, the content of omega 3 was significantly higher in all extruded and extruded–fermented corn by-products, except C_ex14Lu_. The omega 6/omega 3 FA ratio ranged from 11.4 to 48.2 with the highest being for C_conLpa_, and the lowest being for C_ex18Lc_. The decrease in omega 6/omega 3 ratio is desirable for the prevention of cardiovascular diseases, diabetes, obesity, and cancer but there are no recommended specific values [56]. Some fermented samples (C_conLpl_, C_conLu_, C_conLc_, C_conLpa_, and C_ex18Lpl_) contained a very small amount (lower than 0.08%) of trans FA.

Availability of data is limited to being on the effect of fermentation and extrusion on FA profile of corn by-products. Wani et al. [57] reported that FA composition, including PFA and MFA content, of corn-based snacks, was not significantly affected by extrusion. Contrarily, Ramos Diaz et al. [58] found that after extrusion, the content of palmitic, linoleic, oleic, and linolenic acid was reduced in corn-based extrudates, compared to those non-extruded. The slight loss of some lipids during extrusion occurs due to the high temperature, which could also cause the essential reduction in PFA stability, and the formation of amylose–lipid complexes [37,58]. Lower conditions of temperature and moisture in the extrusion process increase the stability of PFA in extruded products during storage [14]. Changes in the FA profile after fermentation could be attributed to the lipolytic activity of LAB [59]. In addition, LAB abilities to generate FA and modify their saturation and desaturation are reported [60].

### 3.5. Antimicrobial Characteristics of Corn By-Products

Diameter inhibition zones (DIZ) of the non-treated and treated corn by-products against pathogenic and opportunistic microorganisms are given in Table 6. Fermented corn by-products C_conLpl_, C_conLu_, and C_conLpa_ displayed inhibition properties against 3 of the 10 tested pathogenic/opportunistic bacterial strains: *Acinetobacter johnsonii, Staphylococcus aureus*, *Aeromonas veronii*. Sample C_conLc_ showed inhibition properties only against *Acinetobacter johnsonii* and *Staphylococcus aureus*. Most of samples, except C_con_, C_ex14_, C_ex16_, C_ex18_, C_ex18Lpl_, C_ex18Lpa_, showed inhibition properties against *Acinetobacter johnsonii* (DIZ of 12.5 mm on average). No efficiency in inhibiting *Salmonella enterica Infantis*, *E. coli*, *Bacillus pseudomycoides*, *Cronobacter sakazakii*, *Hafnia alvei*, *Enterococcus durans*, *Kluyvera cryocrescens* was observed by any of the samples.

The natural bio preservatives in foods and feed or their ingredients not only improve the microbiological safety of these products but are a sustainable approach to protect human or animal health [61]. The antimicrobial effects of fermented corn by-products are highly related to the presence of LAB and their active metabolites [62]. In the fermentable substrate, LAB can synthesize several or more antimicrobial compounds, including bacteriocins, bacteriocin like substances (BLIS), organic acids (lactic, acetic, and propionic acids), acetoin, hydrogen peroxide, acetaldehyde, carbon dioxide, and diacetyl [63]. In order to suspend the growth of pathogenic bacteria, sufficient concentrations of antimicrobial metabolites should be released by LAB and that happens when particular levels of LAB are reached in fermented substrate [64]. However, the composition of substrates (carbohydrates, amino acids, vitamins, fatty acids, and minerals) and fermentation conditions (pH, temperature, aeration, and agitation) could strongly affect the growth of LAB, as well as the accumulation and profile of antimicrobial compounds excreted by LAB [61,62]. Our previous study showed that in this study used *L. plantarum* LUHS122, *L. uvarum* LUHS245, *L. casei* LUHS210, and *L. paracasei* LUHS244 displayed good inhibition properties against pathogenic and opportunistic bacterial strains (*Klebsiella pneumoniae*, *Salmonella enterica* 24 SPn06, *Pseudomonas aeruginosa* 17-331, *Acinetobacter baumanni* 17-380, *Proteus mirabilis*, MRSA M87fox-MRSA–Methicillin-resistant, *Enterococcus faecalis* 86, *Enterococcus faecium* 103, *Bacillus cereus* 18 01, 10–*Streptococcus mutans*, *Enterobackter cloacae*, 12–*Citrobacter freundii*, *Staphylococcus epidermidis*, *Staphylococcus haemolyticus*, *Pastaurella multocida*) [15].

The antifungal activities of the non-treated and treated corn by-products against the species of *Aspergillus niger*, *Memnoniella echinate*, *Chrysosporium merdarium*, *Aspergillus fumigatus*, *Trichoderma viride*, *Rhizopus*, *Fusarium nivale*, *Penicillium viridicatum*, *Aspergillus versatile*, and *Aspergillus ferenczii* were tested and the results are given in Table 7. Delay of Rhizopus spore formation was obtained with extruded and fermented samples of C_ex14Lu_, C_ex14Lc_, C_ex14Lpa_, C_ex14Lpl_, C_ex18Lu_, C_ex18Lc_, and C_ex18Lpa_. A delay of Aspergillus fumigatus spore formation was found with the C_ex14Lpl_ sample. However, the lack of inhibitory ability was detected for the rest of the samples against all tested moulds.

The antifungal activity of extruded and fermented corn by-products could result from the synergistic activities of several antifungal metabolites of LAB, for which profile and concentrations depend on strain, species, as well as on LAB growth conditions (availability of nutrients, temperature, pH, atmosphere, and viscosity) [63,65]. Antifungal metabolites include organic acids (lactic acid, acetic acid, 3-phenyllactic acid, etc.), fatty acids (3-hydroxydecanoic acid, ricinoleic acid, decanoic acid, etc.), cyclic dipeptides, reuterin, hydrogen peroxide, and diacetyl [66]. In this study used LAB strains already showed antifungal properties against *Aspergillus fischeri*, *Aspergillus nidulans*, *Penicillium oxalicum*, *Penicillium funiculosum*, *Fusarium poae*, *Alternaria alternate*, and *Fusarium graminearum* [15].

## 4. Conclusions

The economic efficiency and environmentally friendly production are important aspects in the sustainable valorization of cereals processing by-products, and this is still challenging. The valorization of corn processing by-products could be designed in a more appropriate and sustainable manner by using whole by-product conversion, and by combining extrusion and fermentation processes, as the latter are common and economically efficient processes in the food and feed industry. This study indicated that fermentation with antimicrobial properties possessing LAB strains or the combined technique of extrusion and fermentation improved the microbiological safety of corn by-products. The latter technique increased the content of certain amino acids (e.g., valine, methionine) in most of the samples. Such biogenic amines as cadaverine and spermidine were not found after treatments of corn by-products, while the lowest content of biogenic amines was found in extruded–fermented (with *L. paracasei*-LUHS244, moisture 18%) samples. Applied treatments affected the content of most fatty acids. The level of omega 3 was significantly higher in extruded and extruded–fermented corn by-products. However, the contents of saturated, monounsaturated, and polyunsaturated fatty acids were similar between most of the samples. Corn by-products fermented with *L. plantarum*-LUHS122, *L. uvarum*-LUHS245, and *L. paracasei*-LUHS244 showed antibacterial activity against *Acinetobacter johnsonii, Staphylococcus aureus*, *Aeromonas veronii*. Extruded (14% moisture) and fermented with *L. plantarum*-LUHS122 corn by-products inhibited *Rhizopus* and *Aspergillus fumigatus*. In sum, combining extrusion and fermentation processes for corn by-product valorization can improve certain parameters and properties of these products, and they can be recommended as safer and more nutritious ingredients for food and feed production.

## Figures and Tables

**Table 1:** Acidity parameters of non-treated and treated corn by-products.

Corn By-Product Samples	pH		TTA, °N		Lactic Acid Isomers Content, after 24 h of Fermentation, g/100 g	
	Fermentation Time, h				L (+)	D (−)
	0	24	0	24		
C_con_	6.05 ± 0.01 a	-	0.1 ± 0.1 a	-	0.075 ± 0.008 a	0.022 ± 0.003 a
C_conLpl_	-	3.34 ± 0.02 a	-	4.5 ± 0.2 g	0.327 ± 0.045 c	0.600 ± 0.133 f
C_conLu_	-	3.35 ± 0.05 a	-	3.8 ± 0.1 e	0.274 ± 0.028 c	0.456 ± 0.037 e
C_conLc_	-	3.30 ± 0.03 a	-	4.6 ± 0.2 g	0.310 ± 0.031 c	0.537 ± 0.12 e
C_conLpa_	-	3.44 ± 0.02 b	-	4.0 ± 0.2 f	0.360 ± 0.043 d	0.321 ± 0.027 d
C_ex14_	6.4 ± 0.02 b	-	0.2 ± 0.1 a	-	0.048 ± 0.011 a	0.017 ± 0.002 a
C_ex14Lpl_	-	3.67 ± 0.02 e	-	4.6 ± 0.2 g	0.261 ± 0.059 c	0.504 ± 0.086 e
C_ex14Lu_	-	3.55 ± 0.01 c	-	3.7 ± 0.1 e	0.296 ± 0.043 c	0.409 ± 0.059 e
C_ex14Lc_	-	3.53 ± 0.01 c	-	4.5 ± 0.3 g	0.302 ± 0.029 c	0.445 ± 0.072 e
C_ex14Lpa_	-	3.55 ± 0.02 c	-	3.9 ± 0.3 f	0.362 ± 0.061 d	0.337 ± 0.038 d
C_ex16_	6.38 ± 0.03 b	-	0.2 ± 0.1 a	-	0.124 ± 0.01 a	0.045 ± 0.007 b
C_ex16Lpl_	-	3.71 ± 0.01 e	-	3.4 ± 0.1 e	0.188 ± 0.033 b	0.196 ± 0.04 c
C_ex16Lu_	-	3.69 ± 0.01 e	-	3.6 ± 0.2 e	0.284 ± 0.059 c	0.351 ± 0.065 d
C_ex16Lc_	-	3.60 ± 0.02 d	-	2.9 ± 0.1 d	0.305 ± 0.046 c	0.435 ± 0.05 e
C_ex16Lpa_	-	3.71 ± 0.01 e	-	2.6 ± 0.2 c	0.333 ± 0.029 c	0.281 ± 0.06 d
C_ex18_	6.43 ± 0.01 b	-	0.2 ± 0.1 a	-	0.058 ± 0.01 a	0.01 ± 0.001 a
C_ex18Lpl_	-	3.67 ± 0.01 e	-	5.5 ± 0.1 h	0.221 ± 0.049 b	0.251 ± 0.023 d
C_ex18Lu_	-	3.7 ± 0.02 e	-	2.3 ± 0.1 b	0.169 ± 0.035 b	0.156 ± 0.031 c
C_ex18Lc_	-	3.68 ± 0.01 e	-	2.5 ± 0.1 c	0.241 ± 0.042 b	0.208 ± 0.027 c
C_ex18Lpa_	-	4.25 ± 0.01 f	-	1.8 ± 0.2 a	0.095 ± 0.014 a	0.056 ± 0.008 b

TTA—total titratable acidity; C—corn by-product samples; con—control samples (non-extruded, non-fermented); Lpl, Lu, Lc, Lpa—fermented with *L. plantarum*, *L. uvarum*, *L. casei*, *L. paracasei* strains, respectively; ex—extruded samples; 14, 16, 18—moisture content of the corn by-product samples; nd—not analyzed. Data are represented as means (*n* = 3) ± SE. a–h—mean values within a column denoted with different letters are significantly different (*p* ≤ 0.05).

**Table 2:** Microbiological parameters of the corn by-products.

Corn By- Product Samples	Lactic Acid Bacteria Count	Mould and Yeast Count	Total Bacteria Count	Total Enterobacteria Count
	log_10_CFU/g			
C_con_	5.41 ± 0.11 b	4.11 ± 0.05 b	8.45 ± 0.13 d	6.06 ± 0.06 d
C_conLpl_	7.72 ± 0.09 d	3.72 ± 0.07 a	8.32 ± 0.09 b	5.83 ± 0.07 b
C_conLu_	7.13 ± 0.14 c	3.80 ± 0.06 a	8.08 ± 0.08 a	5.71 ± 0.10 b
C_conLc_	9.39 ± 0.08 k	3.72 ± 0.07 a	8.40 ± 0.09 d	5.99 ± 0.11 c
C_conLpa_	9.19 ± 0.15 i	4.02 ± 0.05 b	8.24 ± 0.09 b	6.01 ± 0.07 c
C_ex14_	5.11 ± 0.06 a	4.45 ± 0.08 f	8.90 ± 0.05 h	6.72 ± 0.11 h
C_ex14Lpl_	7.87 ± 0.15 d	4.21 ± 0.08 c	8.33 ± 0.08 b	6.11 ± 0.10 f
C_ex14Lu_	8.68 ± 0.10 g	3.99 ± 0.07 b	8.38 ± 0.10 c	5.89 ± 0.04 b
C_ex14Lc_	8.32 ± 0.10 f	4.12 ± 0.04 b	8.69 ± 0.05 f	5.77 ± 0.09 b
C_ex14Lpa_	8.91 ± 0.09 g	4.34 ± 0.08 e	8.78 ± 0.09 g	6.04 ± 0.05 d
C_ex16_	5.52 ± 0.10 b	5.21 ± 0.06 j	8.41 ± 0.10 d	6.24 ± 0.07 g
C_ex16Lpl_	9.34 ± 0.13 j	4.92 ± 0.09 h	9.03 ± 0.11 i	5.91 ± 0.05 b
C_ex16Lu_	9.11 ± 0.13 i	5.16 ± 0.11 j	8.80 ± 0.09 g	5.8 ± 0.05 b
C_ex16Lc_	9.22 ± 0.12 i	4.84 ± 0.09 h	8.47 ± 0.16 d	5.45 ± 0.11 a
C_ex16Lpa_	8.96 ± 0.12 h	5.03 ± 0.07 i	8.21 ± 0.11 b	6.11 ± 0.12 f
C_ex18_	5.52 ± 0.07 b	4.63 ± 0.09 g	8.63 ± 0.09 e	6.07 ± 0.09 e
C_ex18Lpl_	9.11 ± 0.18 i	4.56 ± 0.08 g	9.10 ± 0.10 j	5.32 ± 0.10 a
C_ex18Lu_	8.64 ± 0.11 g	4.22 ± 0.05 d	8.12 ± 0.05 a	5.93 ± 0.12 b
C_ex18Lc_	9.02 ± 0.13 i	4.45 ± 0.08 f	8.32 ± 0.07 b	5.99 ± 0.11 c
C_ex18Lpa_	8.13 ± 0.11 e	3.99 ± 0.05 b	7.94 ± 0.05 a	6.01 ± 0.11 c

CFU—colony forming units; C—corn by-product samples; con—control samples (non-extruded, non-fermented); Lpl, Lu, Lc, Lpa—fermented with *L. plantarum*-LUHS122, *L. uvarum*-LUHS245, *L. casei*-LUHS210, and *L. paracasei*-LUHS244 strains, respectively; ex—extruded samples; 14, 16, 18—moisture content of the corn by-product samples. Data are represented as means (*n* = 5) ± SE. a–k—mean values within a column denoted with different letters are significantly different (*p* ≤ 0.05).

**Table 3:** Essential amino acids mass concentration (g/100 g) in corn by-products.

Corn By- Product Samples	His	Thr	Val	Met	Trp	Phe	Ile	Leu	Lys
C_con_	0.080 ± 0.007 d	0.15 ± 0.02 d	0.22 ± 0.03 b	0.060 ± 0.011 b	0.17 ± 0.02 b	0.080 ± 0.016 d	0.36 ± 0.06 d	<LOD	<LOD
C_conLpl_	0.11 ± 0.02 e	0.17 ± 0.03 d	0.22 ± 0.03 b	0.030 ± 0.005 a	0.19 ± 0.02 b	0.090 ± 0.011 e	0.40 ± 0.04 d	0.010 ± 0.001 a	<LOD
C_conLu_	0.110 ± 0.012 e	0.18 ± 0.03 d	0.25 ± 0.04 b	0.050 ± 0.004 a	0.21 ± 0.04 b	0.100 ± 0.010 e	0.41 ± 0.07 d	0.020 ± 0.003 b	<LOD
C_conLc_	0.090 ± 0.015 d	0.17 ± 0.03 d	0.21 ± 0.02 b	0.030 ± 0.006 a	0.18 ± 0.03 b	0.090 ± 0.012 e	0.39 ± 0.06 d	0.010 ± 0.001 a	<LOD
C_conLpa_	0.110 ± 0.010 e	0.170 ± 0.027 d	0.21 ± 0.03 b	0.030 ± 0.006 a	0.190 ± 0.019 b	0.090 ± 0.017 e	0.43 ± 0.04 d	0.010 ± 0.001 a	<LOD
C_ex14_	0.060 ± 0.005 c	0.110 ± 0.010 c	0.15 ± 0.03 a	0.030 ± 0.006 a	0.130 ± 0.018 a	0.060 ± 0.006 d	0.26 ± 0.03 c	<LOD	<LOD
C_ex14Lpl_	0.100 ± 0.018 e	0.190 ± 0.026 e	0.29 ± 0.03 c	0.070 ± 0.006 b	0.20 ± 0.02 b	0.110 ± 0.016 e	0.41 ± 0.08 d	0.020 ± 0.003 b	<LOD
C_ex14Lu_	0.070 ± 0.008 d	0.130 ± 0.026 c	0.180 ± 0.015 a	0.040 ± 0.007 a	0.14 ± 0.03 a	0.060 ± 0.006 d	0.27 ± 0.05 c	0.010 ± 0.001 a	<LOD
C_ex14Lc_	0.070 ± 0.013 d	0.120 ± 0.018 c	0.18 ± 0.03 a	0.040 ± 0.005 a	0.13 ± 0.02 a	0.050 ± 0.006 c	0.25 ± 0.03 c	<LOD	<LOD
C_ex14Lpa_	0.060 ± 0.005 c	0.120 ± 0.015 c	0.180 ± 0.018 a	0.040 ± 0.008 a	0.140 ± 0.019 b	0.060 ± 0.009 d	0.25 ± 0.03 c	<LOD	<LOD
C_ex16_	0.050 ± 0.005 c	0.100 ± 0.012 c	0.22 ± 0.02 b	0.13 ± 0.02 c	0.120 ± 0.018 a	0.040 ± 0.005 c	0.18 ± 0.03 b	<LOD	<LOD
C_ex16Lpl_	0.050 ± 0.005 c	0.090 ± 0.013 b	0.27 ± 0.02 c	0.18 ± 0.02 d	0.12 ± 0.02 a	0.040 ± 0.003 c	0.18 ± 0.03 b	<LOD	<LOD
C_ex16Lu_	0.060 ± 0.006 c	0.130 ± 0.024 d	0.30 ± 0.04 c	0.26 ± 0.03 e	0.16 ± 0.03 b	0.070 ± 0.008 d	0.27 ± 0.03 c	<LOD	<LOD
C_ex16Lc_	0.070 ± 0.007 d	0.130 ± 0.018 d	0.39 ± 0.05 d	0.25 ± 0.02 e	0.160 ± 0.019 b	0.070 ± 0.008 d	0.26 ± 0.04 c	<LOD	<LOD
C_ex16Lpa_	0.060 ± 0.010 c	0.130 ± 0.016 d	0.39 ± 0.05 d	0.31 ± 0.04 e	0.170 ± 0.014 b	0.060 ± 0.012 d	0.25 ± 0.04 c	<LOD	<LOD
C_ex18_	0.020 ± 0.004 a	0.050 ± 0.008 a	0.33 ± 0.07 c	0.36 ± 0.04 f	0.110 ± 0.013 a	0.020 ± 0.003 a	0.110 ± 0.013 a	<LOD	<LOD
C_ex18Lpl_	0.040 ± 0.004 b	0.070 ± 0.012 b	0.43 ± 0.05 d	0.41 ± 0.06 f	0.120 ± 0.017 a	0.030 ± 0.003 b	0.15 ± 0.02 b	<LOD	<LOD
C_ex18Lu_	<LOD	0.050 ± 0.008 a	0.38 ± 0.07 d	0.40 ± 0.06 f	0.100 ± 0.011 a	0.020 ± 0.004 a	0.100 ± 0.018 a	<LOD	<LOD
C_ex18Lc_	0.020 ± 0.002 a	0.050 ± 0.007 a	0.39 ± 0.04 d	0.46 ± 0.05 g	0.100 ± 0.018 a	0.020 ± 0.003 a	0.100 ± 0.015 a	<LOD	<LOD
C_ex18Lpa_	0.030 ± 0.006 a	0.050 ± 0.004 a	0.40 ± 0.05 d	0.49 ± 0.04 g	0.11 ± 0.02 a	0.020 ± 0.003 a	0.100 ± 0.014 a	<LOD	<LOD

His—histidine; Thr—threonine; Val—valine; Met—methionine; Trp—tryptophan; Phe—phenylalanine; Ile—isoleucine; Leu—leucine; Lys—lysine. C—corn by-product samples; con—control samples (non-extruded, non-fermented); Lpl, Lu, Lc, Lpa—fermented with *L. plantarum*-LUHS122, *L. uvarum*-LUHS245, *L. casei*-LUHS210, and *L. paracasei*-LUHS244 strains, respectively; ex—extruded samples; 14, 16, 18—moisture content of the corn by-product samples; <LOD—lower than the limit of detection (1.89 µmol/L for His, 1.36 µmol/L for Leu, 8.73 µmol/L for Lys). Data are represented as means (*n* = 3) ± SE. a–g—mean values within a column denoted with different letters are significantly different (*p* ≤ 0.05).

**Table 4:** Non-essential amino acids mass concentration (g/100g) in corn by-products.

Corn By- Product Samples	Asp	Glu	Ser	Gly	Arg	Ala	Tyr	Cys	Pro
C_con_	0.22 ± 0.04 b	0.69 ± 0.14 d	0.15 ± 0.013 d	0.09 ± 0.011 d	<LOD	0.24 ± 0.03 d	0.12 ± 0.019 e	0.31 ± 0.06 a	0.28 ± 0.02 c
C_conLpl_	0.26 ± 0.05 c	0.74 ± 0.06 d	0.18 ± 0.03 d	0.13 ± 0.019 e	0.05 ± 0.006 d	0.24 ± 0.04 d	0.13 ± 0.018 e	0.24 ± 0.03 a	0.19 ± 0.02 a
C_conLu_	0.27 ± 0.04 c	0.76 ± 0.15 d	0.24 ± 0.03 e	0.14 ± 0.02 e	0.05 ± 0.005 d	0.26 ± 0.02d	0.14 ± 0.02 e	0.33 ± 0.03 a	0.32 ± 0.04 c
C_conLc_	0.26 ± 0.04 c	0.75 ± 0.07 d	0.17 ± 0.018 d	0.12 ± 0.010 e	0.05 ± 0.007 d	0.24 ± 0.05 d	0.13 ± 0.017 e	0.23 ± 0.04 a	0.31 ± 0.05 c
C_conLpa_	0.25 ± 0.03 c	0.79 ± 0.12 d	0.18 ± 0.03 d	0.11 ± 0.019 d	0.03 ± 0.004 c	0.25 ± 0.02 d	0.14 ± 0.015 e	0.22 ± 0.04 a	0.23 ± 0.03 b
C_ex14_	0.18 ± 0.03 b	0.52 ± 0.10 c	0.12 ± 0.013 c	0.08 ± 0.012 d	<LOD	0.17 ± 0.02 b	0.09 ± 0.008 d	0.20 ± 0.03 a	0.29 ± 0.05 c
C_ex14Lpl_	0.30 ± 0.03 d	0.83 ± 0.14 d	0.22 ± 0.04 e	0.16 ± 0.03 f	0.08 ± 0.014 e	0.28 ± 0.03 d	0.14 ± 0.03 e	0.37 ± 0.05 a	0.31 ± 0.03 c
C_ex14Lu_	0.20 ± 0.02 b	0.55 ± 0.10 c	0.14 ± 0.03 c	0.11 ± 0.02 d	0.02 ± 0.002 b	0.19 ± 0.03 c	0.09 ± 0.017 d	0.23 ± 0.04 a	0.22 ± 0.03 b
C_ex14Lc_	0.19 ± 0.04 b	0.53 ± 0.10 c	0.11 ± 0.011 c	0.09 ± 0.009 d	<LOD	0.18 ± 0.022 c	0.09 ± 0.016 d	0.27 ± 0.03 a	0.25 ± 0.04 c
C_ex14Lpa_	0.19 ± 0.02 b	0.52 ± 0.06 c	0.12 ± 0.019 c	0.10 ± 0.016 d	<LOD	0.18 ± 0.016 c	0.08 ± 0.012 c	0.26 ± 0.04 a	0.28 ± 0.06 c
C_ex16_	0.15 ± 0.02 a	0.41 ± 0.06 b	0.12 ± 0.013 c	0.07 ± 0.012 c	<LOD	0.14 ± 0.014 b	0.07 ± 0.011 c	0.53 ± 0.05 b	0.21 ± 0.02 b
C_ex16Lpl_	0.14 ± 0.02 a	0.42 ± 0.06 b	0.08 ± 0.014 b	0.06 ± 0.007 c	<LOD	0.14 ± 0.018 b	0.06 ± 0.010 c	0.69 ± 0.07 c	0.23 ± 0.03 b
C_ex16Lu_	0.20 ± 0.04 b	0.59 ± 0.05 c	0.13 ± 0.019 c	0.09 ± 0.013 d	<LOD	0.2 ± 0.04 c	0.10 ± 0.009 d	0.82 ± 0.14 c	0.24 ± 0.04 b
C_ex16Lc_	0.21 ± 0.03 b	0.60 ± 0.06 c	0.13 ± 0.03 c	0.10 ± 0.018 d	0.01 ± 0.002 a	0.21 ± 0.03 c	0.09 ± 0.009 d	0.96 ± 0.18 d	0.30 ± 0.04 c
C_ex16Lpa_	0.20 ± 0.03 b	0.59 ± 0.08 c	0.13 ± 0.02 c	0.09 ± 0.008 d	<LOD	0.20 ± 0.02 c	0.10 ± 0.012 d	1.05 ± 0.14 d	0.33 ± 0.06 c
C_ex18_	0.09 ± 0.011 a	0.26 ± 0.05 a	0.04 ± 0.005 a	0.02 ± 0.003 a	<LOD	0.09 ± 0.014 a	0.04 ± 0.007 b	1.25 ± 0.13 d	0.19 ± 0.02 a
C_ex18Lpl_	0.13 ± 0.016 a	0.35 ± 0.03 b	0.06 ± 0.008 a	0.05 ± 0.010 c	<LOD	0.13 ± 0.02 b	0.05 ± 0.006 b	1.40 ± 0.13 e	0.20 ± 0.04 a
C_ex18Lu_	0.09 ± 0.015 a	0.25 ± 0.05 a	0.04 ± 0.004 a	0.03 ± 0.003 b	<LOD	0.09 ± 0.015 a	0.03 ± 0.002 a	1.47 ± 0.12 e	0.18 ± 0.03 a
C_ex18Lc_	0.09 ± 0.008 a	0.24 ± 0.04 a	0.04 ± 0.007 a	0.03 ± 0.005 b	<LOD	0.09 ± 0.008 a	0.03 ± 0.006 a	1.62 ± 0.17 e	0.16 ± 0.03 a
C_ex18Lpa_	0.09 ± 0.015 a	0.26 ± 0.03 a	0.03 ± 0.005 a	0.04 ± 0.005 b	<LOD	0.09 ± 0.009 a	0.03 ± 0.006 a	1.69 ± 0.25 e	0.17 ± 0.017 a

Asp—aspartic acid; Glu—glutamine; Asn—asparagine; Ser—serine; Gly—glycine; Arg—arginine; Ala—alanine; Tyr—tyrosine; Cys—cysteine; Pro—proline. C—corn by-product samples; con—control samples (non-extruded, non-fermented); Lpl, Lu, Lc, Lpa—fermented with *L. plantarum*-LUHS122, *L. uvarum*-LUHS245, *L. casei*-LUHS210, and *L. paracasei*-LUHS244 strains, respectively; ex—extruded samples; 14, 16, 18—moisture content of the corn by-product samples; <LOD—lower than the limit of detection (6.78 µmol/L for Arg). Data are represented as means (*n* = 3) ± SE. a–f—mean values within a column denoted with different letters are significantly different (*p* ≤ 0.05).

**Table 5:** The fatty acids percentage profile of the non-treated and treated corn by-products.

Corn By- Product Samples	Saturated	Mono- Unsaturated	Poly- Unsaturated	Trans	Omega 3	Omega 6	Omega 9
C_con_	13.4 ± 1.7 a	33.4 ± 6.5 a	53.2 ± 9.9 b	nd	1.39 ± 0.14 a	51.8 ± 9.7 a	33.2 ± 7.0 a
C_conLpl_	12.9 ± 1.5 a	33.4 ± 4.6 a	53.7 ± 5.3 b	0.010 ± 0.001 a	1.15 ± 0.26 a	52.6 ± 5.3 a	33.3 ± 4.2 a
C_conLu_	13.0 ± 1.9 a	33.5 ± 4.5 a	53.4 ± 11.5 b	0.010 ± 0.002 a	1.23 ± 0.10 a	52.2 ± 7.4 a	33.4 ± 5.4 a
C_conLc_	13.0 ± 2.1 a	33.3 ± 3.9 a	53.7 ± 9.2 b	0.010 ± 0.001 a	1.22 ± 0.12 a	52.5 ± 5.7 a	33.2 ± 6.1 a
C_conLpa_	12.8 ± 1.2 a	33.5 ± 4.0 a	53.6 ± 8.5 b	0.010 ± 0.001 a	1.09 ± 0.21 a	52.5 ± 11.7 a	33.4 ± 6.5 a
C_ex14_	14.2 ± 2.9 a	33.2 ± 5.0 a	52.6 ± 4.7 b	nd	1.67 ± 0.30 b	50.9 ± 7.6 a	33.0 ± 2.7 a
C_ex14Lpl_	12.9 ± 2.6 a	33.4 ± 4.7 a	53.7 ± 6.1 b	nd	1.85 ± 0.31 b	51.9 ± 7.1 a	33.2 ± 3.7 a
C_ex14Lu_	13.3 ± 2.1 a	33.7 ± 3.0 a	53.1 ± 6.5 b	nd	1.34 ± 0.23 a	51.8 ± 10.7 a	33.4 ± 3.8 a
C_ex14Lc_	17.1 ± 2.8 b	33.1 ± 7.0 a	49.9 ± 7.3 b	nd	1.95 ± 0.38 b	47.9 ± 7.9 a	32.8 ± 6.6 a
C_ex14Lpa_	15.0 ± 2.9 a	33.7 ± 2.8 a	51.3 ± 9.0 b	nd	1.68 ± 0.29 b	49.6 ± 4.0 a	33.6 ± 3.1 a
C_ex16_	14.0 ± 2.7 a	33.3 ± 7.0 a	52.7 ± 11.4 b	nd	1.73 ± 0.32 b	51.0 ± 7.6 a	33.2 ± 5.5 a
C_ex16Lpl_	16.0 ± 3.0 a	33.1 ± 5.4 a	50.9 ± 4.5 b	nd	2.12 ± 0.40 b	48.7 ± 6.1 a	33.0 ± 6.3 a
C_ex16Lu_	16.1 ± 2.7 a	33.9 ± 4.6 a	50.0 ± 4.2 a	nd	1.98 ± 0.27 b	48.0 ± 8.4 a	33.7 ± 2.8 a
C_ex16Lc_	14.1 ± 1.8 a	33.6 ± 2.8 a	52.3 ± 4.5 b	nd	1.77 ± 0.17 b	50.5 ± 11.5 a	33.4 ± 4.7 a
C_ex16Lpa_	14.8 ± 1.6 a	34.4 ± 2.8 a	50.8 ± 8.0 b	nd	1.7 ± 0.37 b	49.1 ± 5.0 a	34.3 ± 4.1 a
C_ex18_	14.3 ± 2.9 a	33.5 ± 5.4 a	52.3 ± 9.3 b	nd	1.84 ± 0.21 b	50.5 ± 6.7 a	33.2 ± 4.4 a
C_ex18Lpl_	20.7 ± 3.6 b	32.9 ± 2.7 a	46.4 ± 7.6 b	0.080 ± 0.018 b	1.99 ± 0.43 b	44.4 ± 9.0 a	32.5 ± 5.1 a
C_ex18Lu_	15. 9 ± 3.4 a	34.1 ± 4.0 a	50.0 ± 11.4 b	nd	3.24 ± 0.46 d	46.8 ± 7.6 a	33.9 ± 3.1 a
C_ex18Lc_	20.3 ± 4.6 b	34.8 ± 3.1 a	44.9 ± 4.7 b	nd	3.61 ± 0.48 d	41.3 ± 8.4 a	34.3 ± 4.1 a
C_ex18Lpa_	15.0 ± 1.4 a	33.1 ± 7.3 a	51.9 ± 4.7 b	nd	2.54 ± 0.20 c	49.3 ± 8.7 a	33.0 ± 5.1 a

C—corn by-product samples; con—control samples (non-extruded, non-fermented); Lpl, Lu, Lc, Lpa—fermented with *L. plantarum*-LUHS122, *L. uvarum*-LUHS245, *L. casei*-LUHS210, and *L. paracasei*-LUHS244 strains, respectively; ex—extruded samples; 14, 16, 18—moisture content of the corn by-product samples; nd—not detectable. Data are represented as means (*n* = 3) ± SE. a–d—mean values within a column denoted with different letters are significantly different (*p* ≤ 0.05).

**Table 6:** Antibacterial properties of non-treated and treated corn by-products.

Corn By- Product Samples	Pathogens			
	*Staphylococcus aureus* LT 102	*Aeromonas veronii* LT 105	*Acinetobacter johnsonii* LT 110	
	Diameter of Inhibition Zones, mm			
C_con_	nd	nd	nd	
C_conLpl_	11.0 ± 0.2 c	12.3 ± 0.3 c	15.3 ± 0.1 g	
C_conLu_	9.2 ± 0.1 a	9.6 ± 0.2 b	11.2 ± 0.4 c	
C_conLc_	9.1 ± 0.1 a	nd	9.4 ± 0.3 b	
C_conLpa_	10.3 ± 0.4 b	9.0 ± 0.4 a	12.7 ± 0.2 d	
C_ex14_	nd	nd	nd	
C_ex14Lpl_	nd	nd	11.8 ± 0.5 c	
C_ex14Lu_	nd	nd	12.3 ± 0.3 d	
C_ex14Lc_	nd	nd	12.5 ± 0.6 d	
C_ex14Lpa_	nd	nd	12.2 ± 0.2 d	
C_ex16_	nd	nd	nd	
C_ex16Lpl_	nd	nd	14.3 ± 0.3 f	
C_ex16Lu_	nd	nd	14.4 ± 0.2 f	
C_ex16Lc_	nd	nd	7.20 ± 0.10 a	
C_ex16Lpa_	nd	nd	13.6 ± 0.3 e	
C_ex18_	nd	nd	nd	
C_ex18Lpl_	nd	nd	nd	
C_ex18Lu_	nd	nd	12.2 ± 0.3 d	
C_ex18Lc_	nd	nd	15.6 ± 0.2 g	
C_ex18Lpa_	nd	nd	nd	
	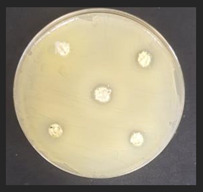	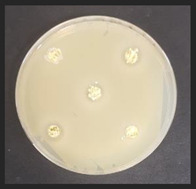	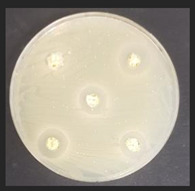	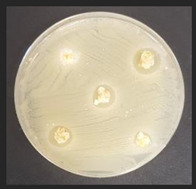
	Against *Staphylococcus aureus*	Against *Aeromonas veronii*	Against *Acinetobacter johnsonii*	

C—corn by-product samples; con—control samples (non-extruded, non-fermented); Lpl, Lu, Lc, Lpa—fermented with *L. plantarum*-LUHS122, *L. uvarum*-LUHS245, *L. casei*-LUHS210, and *L. paracasei*-LUHS244 strains, respectively; ex—extruded samples; 14, 16, 18—moisture content of the corn by-product samples. nd—not detected. Data are represented as means (*n* = 5) ± SE. a–g—mean values within a column denoted with different letters are significantly different (*p* ≤ 0.05).

**Table 7:** Antifungal properties of non-treated and treated corn by-products.

Fungi	Corn By-Product Samples																			
	C_con_	C_conLpl_	C_conLu_	C_conLc_	C_conLpa_	C_ex14_	C_ex14Lpl_	C_ex14Lu_	C_ex14Lc_	C_ex14Lpa_	C_ex16_	C_ex16Lpl_	C_ex16Lu_	C_ex16Lc_	C_ex16Lpa_	C_ex18_	C_ex18Lpl_	C_ex18Lu_	C_ex18Lc_	C_ex18Lpa_
*Aspergillus fumigatus*	-	-	-	-	-	-	+	-	-	-	-	-	-	-	-	-	-	-	-	-
*Rhizopus*	-	-	-	-	-	-	+	+	+	+	-	-	-	-	-	-	-	+	+	+

C—corn by-product samples; con—control samples (non-extruded, non-fermented); Lpl, Lu, Lc, Lpa—fermented with *L. plantarum*-LUHS122, *L. uvarum*-LUHS245, *L. casei*-LUHS210, and *L. paracasei*-LUHS244 strains, respectively; ex—extruded samples; 14, 16, 18—moisture content of the corn by-product samples. Interpretation of inhibition of fungi by the corn by-products: (-) no inhibition, (+) delay of spore formation.

## Data Availability

The data presented in this study are available on request from the corresponding author.

## Supplementary Materials

The following supporting information can be downloaded at: https://www.mdpi.com/article/10.3390/plants11223080/s1. Supplementary Data S1: Description of Methods; Supplementary Data S2. Table S1: The fatty acids profile of the corn by-products; Supplementary Data S3. Table S2: Limit of detection values (LOD) for analyzed fatty acids.
