# Supplementary material for: Combined Thermomechanical–Biological Treatment for Corn By-Product Valorization into Added-Value Food (Feed) Material
Source: Plants (Basel). 2022 Nov 14;11(22):3080. doi: 10.3390/plants11223080 (PMC9696026; doi:10.3390/plants11223080)
Supplement: Supplementary file 1 [file plants-11-03080-s001.zip › Supplementary Data S1. Description of Methods.pdf]

Description of Methods.

Analysis of The Amino Acids Profile and Biogenic Amines Concentration

For amino acids analysis, analytes were extracted from homogenized sample with aqueous 0.1 M HCl solution and dansylation were performed according to the method of Hua-Lin Cai et al. [17], with some modifications. The homogenized sample (~100 mg) was weighed into a 1.5 mL tube, and analytes were extracted with 1 mL of aqueous 0.1 M HCl solution by shaking for 1 h. The resultant mixture was centrifuged at 12,000 rpm for 5 minutes. For derivatization, 50 µL of the resultant supernatant was mixed with 100 µL of 100 mg/L diaminoheptane (as an internal standard) and diluted to 500 µL with 0.1 M HCl solution. The resultant mixture was alkalised by addition of 40 µL of 2 M NaOH and 70 µL of the saturated $NaHCO_3$ solution. Derivatization was performed by adding 1 mL of 10 mg/mL dansyl chloride solution in acetonitrile and incubating the resulting mixture at 60 °C for 30 minutes. The reaction mixture was quenched using 50 µL of 25% ammonia solution and filtered through a 0.22 µm membrane filter into the autosampler vial. The concentration of analytes was determined using The Varian ProStar HPLC system (Varian Corp., Palo Alto, California, USA: two ProStar 210 pumps and a ProStar 410 autosampler) and Thermo Scientific LCQ Fleet Ion trap mass detector. For analyte detection, the mass spectrometer was operated in positive-ionisation single-ion monitoring mode for specific ions corresponding to derivatized analytes. The analyte concentration was determined from a calibration curve, which was obtained by derivatizing the analytes at different concentrations. For the separation of derivatives, a Discovery® HS C_{18} column (150 × 4.6 mm, 5 µm; SupelcoTM Analytical, Bellefonte, Pennsylvania, USA) was used. Mobile phase A was 0.1% formic acid in 5% aqueous acetonitrile, and phase B was 0.1% in acetonitrile. A flow rate of 0.3 mL/min was used for the analysis. The injection volume was 10 µL. The analytical gradient was as follows: 0 to 10 min (linear gradient) 15% to 60% B, 10 to 40 min (linear gradient) 60% to 95 % B, 40 to 48 min 95 B, followed by reequilibration for 10 minutes with 15% B (increased to 0.6 mL/min flowrate). The limit of quantification (according to the lowest concentration used for calibration) was 0.02 µmol/g.

Biogenic amines (BA) were analysed according to the method of Ben-Gigirey et al. [18] with some modifications by Bartkiene et al. [19]. Following BAs were analyzed: tryptamine, phenylethylamine, cadaverine, putrescine, histamine, tyramine, spermine, and spermidine.

The standard BA solutions were prepared by dissolving known amounts of each BAs (including internal standard – 1.7-diamino-heptane) in 20 ml of deionized water. Briefly, the extraction of BA in samples (5 g) was done by using 0.4 mol/L perchloric acid. The derivatization of sample extracts and standards was performed using a dansyl chloride solution in acetonitrile (10 mg/ml) as a reagent. The Varian ProStar HPLC system (Varian Corp., Palo Alto, California, USA) was made up of the

following: two ProStar 210 pumps, a ProStar 410 autosampler, a ProStar 325 UV/VIS Detector, and Galaxy software (Agilent, Santa Clara, California, USA) for data processing. For the separation of amines, a Discovery® HS C18 column (150 × 4.6 mm, 5 µm; SupelcoTM Analytical, Bellefonte, Pennsylvania, USA) was used. The eluents were ammonium acetate (A) and acetonitrile (B) and the elution program consisted of a gradient system with a 0.8 ml/min flow rate. The target compounds were identified based on their retention times in comparison to their corresponding standards. Linearity of the calibration curves was established by injecting five concentrations of each biogenic amine standard (0.00–0.28 g/L for tryptamine, phenylethylamine, cadaverine, putrescine, histamine, tyramine, and spermidine; 0.01–1.40 g/L for spermine). Good linearity (R^2: 0.9963–0.9999) was obtained between peak area and analyte concentration.

Determination of Sugars Concentration in Corn By-product Samples

To determine sugar concentrations, 2–3 g of sample was diluted with ~70 mL of distilled/deionized water, heated to 60 °C in a water bath for 15 min, clarified with 2.5 mL Carrez I (85 mM $K_4[Fe(CN)_6]\times3H_2O$) and 2.5 mL Carrez II (250 mM $ZnSO_4\times7H_2O$) solutions, and made up to 100 mL with distilled/deionized water. After 15 min, the samples were filtered through a filter paper and a 0.22 µm nylon syringe filter before analysis. A standard solution of a sugar mixture was prepared by dissolving 0.2 g each of fructose (Sigma-Aldrich, Germany), glucose (Sigma-Aldrich, Germany), sucrose (Sigma-Aldrich, Germany), and maltose (Sigma-Aldrich, Germany) in 100 mL of distilled/deionized water. A 2 mg mL^{-1} standard solution of sugar mixture was prepared following dilution with distilled/deionized water. Sugars concentration analysis was performed by Ultra Performance Liquid Chromatography (UPLC). The UPLC apparatus was a Shimadzu LC-20AD (Shimadzu Corp., Kyoto, Japan) equipped with Evaporative Light Scattering Detector ELSDLTII (Shimadzu Corp., Kyoto, Japan) detector. Chromatographic conditions were as follows: the eluent was a mixture of 75 parts by volume of acetonitrile and 25 parts by volume water, flow rate was 1.2 mL min^{-1}, 20 µL was injected. A YMC-Pack Polyamine II column (250 × 4.6 mm, 5 µm; YMC Co., Ltd., Japan) was used. Column temperature was set at 28 °C. Detection was performed using an ELSDLTII evaporative light scattering detector (Shimadzu Corp., Japan).

Evaluation of Fatty Acids Profile

The fatty acid (FA) composition of the non-treated and treated corn by-products samples was determined using GCMS-QP2010 (Shimadzu, Japan) gas chromatograph with a mass spectrometer. The FA methyl esters (FAME) concentration was determined using a calibration curve and results were expressed as the percentage of total FAME concentration in the sample. The sample was

prepared by homogenizing 1 g of WW sample in 5 mL of 30% (*w*/*v*) NaCl solution. Next, 5 mL of hexane were added. The mixture was shaken on a laboratory shaker for 1 h. The mixture was centrifuged at 4000 rpm. Afterwards 4 mL of the hexane extract were reacted with 300 μL of methylation reagent (2 mol/L of KOH in methanol) by vortexing and shaking using a laboratory shaker for 1 h. The mixture was centrifuged at 4000 rpm and the upper layer was filtered using a 0.22 μm membrane syringe filter and used for the analysis. Capillary Stabilwax-MS column (30 m × 0.25 mm ID × 0.25 μm film thickness. Mass spectrometer operated at full scan mode. Analyte was injected in split mode at 1:60 split ratio. The following parameters were used: MS ion source temperature: 240 °C, MS interface temperature 240 °C, helium (carrier gas) flow: 0,90 mL/min, injector: 240 °C, oven temperature 50 °C (4 min), 10 °C/min o 110 °C (1 min), 15 °C min^{-1} to 160 °C (2 min), 2,5 °C/min to 195 °C (1 min), 2 °C/min to 230 °C (1 min), 2 °C min^{-1} to 240 °C (12 min).

Evaluation of antimicrobial properties

The antibacterial activity of the non-treated and treated corn by-products against a variety of pathogenic and opportunistic bacterial strains (*Salmonella enterica Infantis* LT 101, *Staphylococcus aureus* LT 102, *E. coli* LT 103, *Bacillus pseudomycoi*des LT 104, *Aeromonas veronii* LT 105, *Cronobacter sakazakii* LT 106, *Hafnia alvei* LT 107, *Enterococcus durans* LT 108, *Kluyvera cryocrescens* LT 109, *Acinetobacter johnsonii* LT 110) was evaluated. The pathogenic and opportunistic bacterial strains used were obtained from the Lithuanian University of Health Sciences (Kaunas, Lithuania) collection. The antibacterial activity of the non-treated and treated corn by-products was assessed by measuring the diameter of inhibition zones (DIZ, mm) in agar well diffusion assays. For this purpose, 0.5 McFarland unit density suspension of each pathogenic bacteria strain was inoculated onto the surface of cooled Mueller–Hinton agar (Oxoid, UK) using sterile cotton swabs. Wells of 6 mm in diameter were punched in the agar and filled with the tested by-product. Before the experiment, the corn by-products were diluted with sterile physiological solution (1 g of the by-product diluted with 2 mL of the physiological solution). The average DIZ was calculated from triplicate experiments.

The antifungal activities of the non-treated and treated corn by-products were determined against ten different mold species (*Aspergillus niger, Memnoniella echinate, Chrysosporium merdarium, Aspergillus fumigatus, Trichoderma viride, Rhizopus, Fusarium nivale, Penicillium viridicatum, Aspergillus versatile, and Aspergillus ferenczii*). These molds were previously isolated from grain-based food and were provided by the collection of the Lithuanian University of Health Sciences (Kaunas, Lithuania). All these microorganisms were cultivated on yeast extract, peptone and dextrose

(YEPD) agar medium at 25 °C, in the thermostat for 5 days. The antifungal activity of LAB strains was tested by the agar well diffusion assay [20].
