# Supplementary material for: Combined Thermomechanical–Biological Treatment for Corn By-Product Valorization into Added-Value Food (Feed) Material
Source: Plants (Basel). 2022 Nov 14;11(22):3080. doi: 10.3390/plants11223080 (PMC9696026; doi:10.3390/plants11223080)
Supplement: Supplementary file 1 [file plants-11-03080-s001.zip › Supplementary Data S2. Table S1. Fatty acids ¿C kopija.pdf]

SUPPLEMENTARY MATERIAL

Table S1. The fatty acids percentage profile of the corn by-products.

Fatty acid	Corn by-product samples																			
	C_{con}	C_{conLpl}	C_{conLu}	C_{conLc}	C_{conLpa}	C_{ex14}	$C_{ex14Lpl}$	C_{ex14Lu}	C_{ex14Lc}	$C_{ex14Lpa}$	C_{ex16}	$C_{ex16Lpl}$	C_{ex16Lu}	C_{ex16Lc}	$C_{ex16Lpa}$	C_{ex18}	$C_{ex18Lpl}$	C_{ex18Lu}	C_{ex18Lc}	$C_{ex18Lpa}$
C4:0	< LOD	< LOD	< LOD	< LOD	< LOD	< LOD	< LOD	< LOD	0.07 ± 0.007 b	< LOD	< LOD	0.06 ± 0.008 b	0.08 ± 0.014 b	< LOD	0.03 ± 0.005 a	< LOD	0.17 ± 0.021 c	< LOD	< LOD	< LOD
C6:0	< LOD	< LOD	< LOD	< LOD	< LOD	0.03 ± 0.005 a	< LOD	< LOD	0.07 ± 0.012 b	< LOD	< LOD	0.05 ± 0.004 b	0.07 ± 0.016 b	< LOD	< LOD	< LOD	0.18 ± 0.029 c	< LOD	< LOD	< LOD
C8:0	< LOD	< LOD	0.01 ± 0.002 a	0.01 ± 0.001 a	< LOD	0.03 ± 0.004 b	< LOD	< LOD	0.09 ± 0.011 d	< LOD	< LOD	0.11 ± 0.018 d	0.06 ± 0.006 c	< LOD	0.03 ± 0.004 b	0.05 ± 0.011 c	0.15 ± 0.021 e	< LOD	0.11 ± 0.022 d	< LOD
C10:0	< LOD	< LOD	0.01 ± 0.001 a	0.01 ± 0.002 a	0.01 ± 0.002 a	< LOD	< LOD	< LOD	0.15 ± 0.017 d	0.08 ± 0.016 c	< LOD	0.09 ± 0.009 c	0.16 ± 0.026 d	< LOD	< LOD	0.05 ± 0.006 b	0.29 ± 0.03 e	< LOD	0.14 ± 0.032 d	< LOD
C10:1	< LOD	< LOD	< LOD	< LOD	< LOD	< LOD	< LOD	< LOD	< LOD	< LOD	< LOD	< LOD	< LOD	< LOD	< LOD	< LOD	< LOD	< LOD	< LOD	< LOD
C10:2	< LOD	< LOD	< LOD	< LOD	< LOD	< LOD	< LOD	< LOD	< LOD	< LOD	< LOD	< LOD	< LOD	< LOD	< LOD	< LOD	< LOD	< LOD	< LOD	< LOD
C11:0	< LOD	< LOD	< LOD	< LOD	< LOD	< LOD	< LOD	< LOD	< LOD	< LOD	< LOD	< LOD	< LOD	< LOD	< LOD	< LOD	< LOD	< LOD	< LOD	< LOD
C11:1	< LOD	< LOD	< LOD	< LOD	< LOD	< LOD	< LOD	< LOD	< LOD	< LOD	< LOD	< LOD	< LOD	< LOD	< LOD	< LOD	< LOD	< LOD	< LOD	< LOD
C11:2	< LOD	< LOD	< LOD	< LOD	< LOD	< LOD	< LOD	< LOD	< LOD	< LOD	< LOD	< LOD	< LOD	< LOD	< LOD	< LOD	< LOD	< LOD	< LOD	< LOD
C12:0	0.03 ± 0.004 c	0.02 ± 0.003 b	0.03 ± 0.006 c	0.03 ± 0.006 c	0.01 ± 0.001 a	0.17 ± 0.025 f	0.04 ± 0.008 c	< LOD	0.44 ± 0.054 h	0.26 ± 0.046 g	0.2 ± 0.017 f	0.34 ± 0.064 g	0.2 ± 0.033 f	0.12 ± 0.014 e	0.07 ± 0.015 d	0.11 ± 0.019 e	0.59 ± 0.124 i	0.36 ± 0.037 h	0.37 ± 0.058 h	0.15 ± 0.023 e
C12:1	< LOD	< LOD	< LOD	< LOD	< LOD	< LOD	< LOD	< LOD	< LOD	< LOD	< LOD	< LOD	< LOD	< LOD	< LOD	< LOD	< LOD	< LOD	< LOD	< LOD
C12:2	< LOD	< LOD	< LOD	< LOD	< LOD	< LOD	< LOD	< LOD	< LOD	< LOD	< LOD	< LOD	< LOD	< LOD	< LOD	< LOD	< LOD	< LOD	< LOD	< LOD
C13:0	< LOD	< LOD	< LOD	< LOD	< LOD	< LOD	< LOD	< LOD	< LOD	< LOD	< LOD	< LOD	< LOD	< LOD	< LOD	< LOD	< LOD	< LOD	< LOD	< LOD
C13:1	< LOD	< LOD	< LOD	< LOD	< LOD	< LOD	< LOD	< LOD	< LOD	< LOD	< LOD	< LOD	< LOD	< LOD	< LOD	< LOD	< LOD	< LOD	< LOD	< LOD
C13:2	< LOD	< LOD	< LOD	< LOD	< LOD	< LOD	< LOD	< LOD	< LOD	< LOD	< LOD	< LOD	< LOD	< LOD	< LOD	< LOD	< LOD	< LOD	< LOD	< LOD
C14:0	0.09 ± 0.014 b	0.05 ± 0.007 a	0.08 ± 0.014 b	0.08 ± 0.018 b	0.05 ± 0.007 a	0.22 ± 0.019 c	0.07 ± 0.012 a	0.09 ± 0.018 b	0.67 ± 0.054 g	0.32 ± 0.067 d	0.19 ± 0.02 c	0.51 ± 0.103 f	0.63 ± 0.067 f	0.26 ± 0.026 d	0.19 ± 0.03 c	0.24 ± 0.049 c	1.4 ± 0.284 i	0.43 ± 0.084 e	0.83 ± 0.077 h	0.33 ± 0.058 d
C14:1	< LOD	< LOD	< LOD	< LOD	< LOD	< LOD	< LOD	< LOD	< LOD	< LOD	< LOD	< LOD	< LOD	< LOD	< LOD	< LOD	0.1 ± 0.012	< LOD	< LOD	< LOD

C14:2	< LOD	< LOD	< LOD	< LOD	< LOD	< LOD	< LOD	< LOD	< LOD	< LOD	< LOD	< LOD	< LOD	< LOD	< LOD	< LOD	< LOD	< LOD	< LOD	< LOD
C15:0	< LOD	< LOD	0.01 ± 0.002 a	0.01 ± 0.001 a	0.01 ± 0.001 a	0.03 ± 0.004 b	< LOD	< LOD	0.08 ± 0.009 c	< LOD	< LOD	< LOD	< LOD	< LOD	< LOD	< LOD	0.13 ± 0.021 d	< LOD	< LOD	< LOD
C15:1	< LOD	< LOD	0.01 ± 0.002 a	0.02 ± 0.004 b	< LOD	0.06 ± 0.013 c	< LOD	< LOD	0.08 ± 0.012 c	< LOD	< LOD	< LOD	< LOD	< LOD	< LOD	< LOD	0.11 ± 0.025 d	< LOD	< LOD	< LOD
C15:2	< LOD	< LOD	< LOD	< LOD	< LOD	< LOD	< LOD	< LOD	< LOD	< LOD	< LOD	< LOD	< LOD	< LOD	< LOD	< LOD	< LOD	< LOD	< LOD	< LOD
C16:0	9.81 ± 1.136 a	9.56 ± 1.447 a	9.62 ± 2.125 a	9.59 ± 1.65 a	9.63 ± 0.809 a	10.17 ± 1.034 a	9.61 ± 0.882 a	9.78 ± 1.664 a	11.69 ± 1.091 a	10.64 ± 2.26 a	10.08 ± 1.238 a	11.08 ± 1.854 a	10.97 ± 1.731 a	10.09 ± 1.56 a	10.92 ± 1.363 a	10.14 ± 1.5 a	13.42 ± 1.408 b	11.05 ± 1.684 a	13.59 ± 1.228 b	10.95 ± 1.86 a
C16:1	0.14 ± 0.023 b	0.1 ± 0.009 a	0.12 ± 0.011 a	0.1 ± 0.019 a	0.09 ± 0.02 a	0.14 ± 0.029 b	0.11 ± 0.015 a	0.16 ± 0.033 b	0.19 ± 0.038 b	0.16 ± 0.022 b	0.13 ± 0.028 a	0.16 ± 0.013 b	0.21 ± 0.039 c	0.15 ± 0.017 b	0.14 ± 0.029 a	0.16 ± 0.025 b	0.22 ± 0.027 c	0.21 ± 0.034 c	0.43 ± 0.057 d	0.14 ± 0.031 a
C16:2	< LOD	< LOD	< LOD	< LOD	< LOD	< LOD	< LOD	< LOD	< LOD	< LOD	< LOD	< LOD	< LOD	< LOD	< LOD	< LOD	< LOD	< LOD	< LOD	< LOD
C17:0	0.06 ± 0.01 a	0.06 ± 0.008 a	0.06 ± 0.012 a	0.06 ± 0.009 a	0.06 ± 0.006 a	0.09 ± 0.017 b	0.06 ± 0.013 a	0.07 ± 0.007 a	0.12 ± 0.02 b	0.07 ± 0.008 a	0.09 ± 0.021 a	0.08 ± 0.007 a	0.11 ± 0.022 b	0.08 ± 0.017 a	0.08 ± 0.015 a	0.08 ± 0.013 a	0.16 ± 0.014 c	< LOD	0.15 ± 0.021 c	< LOD
C17:1	0.03 ± 0.007 a	0.03 ± 0.004 a	0.03 ± 0.002 a	0.03 ± 0.006 a	0.03 ± 0.004 a	0.03 ± 0.003 a	0.04 ± 0.007 b	0.05 ± 0.011 b	< LOD	< LOD	< LOD	< LOD	< LOD	0.05 ± 0.006 b	0.03 ± 0.003 a	0.05 ± 0.011 b	< LOD	< LOD	< LOD	< LOD
C17:2	< LOD	< LOD	< LOD	< LOD	< LOD	< LOD	< LOD	< LOD	< LOD	< LOD	< LOD	< LOD	< LOD	< LOD	< LOD	< LOD	< LOD	< LOD	< LOD	< LOD
C18:0	2.65 ± 0.534 a	2.36 ± 0.443 a	2.41 ± 0.286 a	2.4 ± 0.533 a	2.32 ± 0.528 a	2.72 ± 0.392 a	2.4 ± 0.549 a	2.52 ± 0.223 a	2.95 ± 0.259 a	2.8 ± 0.308 a	2.63 ± 0.435 a	2.82 ± 0.284 a	3.01 ± 0.246 a	2.72 ± 0.386 a	2.69 ± 0.38 a	2.83 ± 0.232 a	3.48 ± 0.649 b	3.44 ± 0.714 b	4.35 ± 0.845 b	2.78 ± 0.263 a
C18:1 tr.	< LOD	0.01 ± 0.001 a	0.01 ± 0.002 a	0.01 ± 0.001 a	0.01 ± 0.002 a	< LOD	< LOD	< LOD	< LOD	< LOD	< LOD	< LOD	< LOD	< LOD	< LOD	< LOD	0.08 ± 0.014 b	< LOD	< LOD	< LOD
C18:1	32.91 ± 6.351 a	33.05 ± 4.593 a	33.11 ± 4.656 a	32.95 ± 5.529 a	33.16 ± 6.352 a	32.7 ± 7.234 a	32.95 ± 6.979 a	33.16 ± 6.036 a	32.5 ± 7.253 a	33.32 ± 7.192 a	32.92 ± 2.794 a	32.72 ± 6.851 a	33.44 ± 5.138 a	33.06 ± 7.531 a	33.98 ± 2.781 a	32.97 ± 6.513 a	32.16 ± 3.396 a	33.51 ± 7.167 a	33.7 ± 3.187 a	32.74 ± 7.058 a
C18:2 tr.	< LOD	< LOD	< LOD	< LOD	< LOD	< LOD	< LOD	< LOD	< LOD	< LOD	< LOD	< LOD	< LOD	< LOD	< LOD	< LOD	< LOD	< LOD	< LOD	< LOD
C18:2	51.84 ± 10.745 a	52.58 ± 5.282 a	52.18 ± 10.126 a	52.44 ± 9.034 a	52.49 ± 9.557 a	50.9 ± 11.561 a	51.85 ± 5.119 a	51.7 ± 5.8 a	47.91 ± 10.051 a	49.62 ± 5.513 a	50.94 ± 8.246 a	48.77 ± 9.481 a	48.02 ± 9.109 a	50.44 ± 6.885 a	48.89 ± 6.828 a	50.46 ± 8.265 a	44.28 ± 7.863 a	46.61 ± 9.771 a	41.02 ± 4.912 a	49.34 ± 9.024 a
C20:0	0.48 ± 0.049 a	0.48 ± 0.043 a	0.48 ± 0.085 a	0.48 ± 0.1 a	0.48 ± 0.093 a	0.47 ± 0.095 a	0.49 ± 0.112 a	0.48 ± 0.075 a	0.45 ± 0.076 a	0.47 ± 0.056 a	0.51 ± 0.061 a	0.48 ± 0.048 a	0.48 ± 0.069 a	0.53 ± 0.049 a	0.47 ± 0.087 a	0.49 ± 0.058 a	0.41 ± 0.094 a	0.41 ± 0.052 a	0.46 ± 0.044 a	0.42 ± 0.09 a
C18:3	1.18 ± 0.175 a	1.01 ± 0.174 a	1.04 ± 0.122 a	1.05 ± 0.217 a	0.95 ± 0.213 a	1.46 ± 0.21 b	1.69 ± 0.195 b	1.25 ± 0.141 a	1.81 ± 0.251 b	1.53 ± 0.323 b	1.59 ± 0.15 b	1.98 ± 0.347 b	1.78 ± 0.202 b	1.59 ± 0.147 b	1.57 ± 0.195 b	1.73 ± 0.286 b	1.84 ± 0.273 b	2.89 ± 0.313 c	2.92 ± 0.41 c	2.54 ± 0.262 c
C20:1	0.3 ± 0.037 b	0.2 ± 0.041 a	0.27 ± 0.028 b	0.23 ± 0.022 a	0.24 ± 0.035 a	0.26 ± 0.057 a	0.26 ± 0.046 a	0.27 ± 0.036 a	0.3 ± 0.069 a	0.25 ± 0.054 a	0.24 ± 0.027 a	0.25 ± 0.057 a	0.27 ± 0.035 a	0.3 ± 0.059 b	0.29 ± 0.056 a	0.26 ± 0.048 a	0.3 ± 0.053 b	0.34 ± 0.071 b	0.64 ± 0.083 c	0.25 ± 0.049 a
C18:3	< LOD	< LOD	< LOD	< LOD	< LOD	< LOD	< LOD	< LOD	< LOD	< LOD	< LOD	< LOD	< LOD	< LOD	0.14 ± 0.021	< LOD	< LOD	< LOD	< LOD	< LOD

C18:4	< LOD	< LOD	< LOD	< LOD	< LOD	< LOD	< LOD	< LOD	< LOD	< LOD	< LOD	< LOD	< LOD	< LOD	< LOD	< LOD	< LOD	< LOD	< LOD	< LOD
C21:0	< LOD	0.02 ± 0.003 b	0.01 ± 0.002 a	0.01 ± 0.001 a	0.01 ± 0.002 a	< LOD	< LOD	< LOD	< LOD	< LOD	< LOD	< LOD	< LOD	< LOD	< LOD	< LOD	< LOD	< LOD	< LOD	< LOD
C20:2	< LOD	0.01 ± 0.001 a	0.02 ± 0.004 b	0.01 ± 0.002 a	0.02 ± 0.003 b	< LOD	< LOD	0.04 ± 0.006 c	< LOD	< LOD	< LOD	< LOD	< LOD	< LOD	0.02 ± 0.005 b	< LOD	< LOD	< LOD	< LOD	< LOD
C22:0	0.15 ± 0.022 a	0.15 ± 0.031 a	0.13 ± 0.02 a	0.13 ± 0.023 a	0.13 ± 0.026 a	0.16 ± 0.018 b	0.12 ± 0.017 a	0.15 ± 0.019 a	0.14 ± 0.018 a	0.19 ± 0.019 b	0.14 ± 0.023 a	0.15 ± 0.025 a	0.18 ± 0.039 b	0.15 ± 0.028 a	0.17 ± 0.021 b	0.12 ± 0.017 a	0.16 ± 0.015 b	0.2 ± 0.039 b	0.18 ± 0.031 b	0.2 ± 0.045 b
C20:3	< LOD	< LOD	< LOD	0.01 ± 0.001 a	< LOD	< LOD	< LOD	< LOD	< LOD	< LOD	0.07 ± 0.008 b	< LOD	< LOD	0.1 ± 0.012 c	< LOD	< LOD	0.1 ± 0.012 c	0.2 ± 0.022 d	0.24 ± 0.03 d	< LOD
C22:1	< LOD	< LOD	0.01 ± 0.002 a	0.01 ± 0.001 a	0.01 ± 0.002 a	< LOD	< LOD	< LOD	< LOD	< LOD	< LOD	< LOD	< LOD	< LOD	< LOD	< LOD	< LOD	< LOD	< LOD	< LOD
C20:3	0.12 ± 0.021 b	0.09 ± 0.016 a	0.1 ± 0.017 b	0.09 ± 0.008 b	0.07 ± 0.006 a	0.16 ± 0.036 c	0.1 ± 0.01 b	0.09 ± 0.016 b	0.15 ± 0.016 c	0.14 ± 0.025 c	0.14 ± 0.013 c	0.14 ± 0.019 c	0.2 ± 0.035 d	0.18 ± 0.032 c	0.13 ± 0.012 c	0.12 ± 0.019 b	0.15 ± 0.034 c	0.35 ± 0.044 e	0.31 ± 0.048 e	< LOD
C23:0	< LOD	< LOD	< LOD	< LOD	< LOD	< LOD	< LOD	< LOD	< LOD	< LOD	< LOD	< LOD	< LOD	< LOD	< LOD	< LOD	< LOD	< LOD	< LOD	< LOD
C20:4	< LOD	< LOD	< LOD	0.01 ± 0.002 a	0.01 ± 0.001 a	< LOD	< LOD	< LOD	< LOD	< LOD	< LOD	< LOD	< LOD	< LOD	< LOD	< LOD	< LOD	< LOD	< LOD	< LOD
C22:2	< LOD	< LOD	< LOD	< LOD	0.01 ± 0.002	< LOD	< LOD	< LOD	< LOD	< LOD	< LOD	< LOD	< LOD	< LOD	< LOD	< LOD	< LOD	< LOD	< LOD	< LOD
C24:0	0.13 ± 0.021 a	0.16 ± 0.031 b	0.15 ± 0.019 b	0.15 ± 0.02 a	0.14 ± 0.018 a	0.14 ± 0.031 a	0.14 ± 0.015 a	0.17 ± 0.021 b	0.14 ± 0.028 a	0.14 ± 0.03 a	0.12 ± 0.011 a	0.2 ± 0.017 c	0.13 ± 0.014 a	0.19 ± 0.019 c	0.17 ± 0.037 b	0.15 ± 0.016 b	0.14 ± 0.028 a	nd	0.17 ± 0.02 b	0.16 ± 0.025 b
C20:5	< LOD	< LOD	0.02 ± 0.004	< LOD	< LOD	< LOD	< LOD	< LOD	< LOD	< LOD	< LOD	< LOD	< LOD	< LOD	< LOD	< LOD	< LOD	< LOD	0.2 ± 0.035	< LOD
C24-1	< LOD	< LOD	< LOD	< LOD	0.01 ± 0.001	< LOD	< LOD	< LOD	< LOD	< LOD	< LOD	< LOD	< LOD	< LOD	< LOD	< LOD	< LOD	< LOD	< LOD	< LOD
C22-5	< LOD	< LOD	< LOD	< LOD	< LOD	< LOD	< LOD	< LOD	< LOD	< LOD	< LOD	< LOD	< LOD	< LOD	< LOD	< LOD	< LOD	< LOD	< LOD	< LOD
C22-6	0.1 ± 0.02 b	0.05 ± 0.009 a	0.07 ± 0.01 b	0.06 ± 0.014 a	0.05 ± 0.008 a	0.06 ± 0.005 a	0.06 ± 0.008 a	< LOD	< LOD	< LOD	< LOD	< LOD	< LOD	< LOD	< LOD	< LOD	< LOD	< LOD	0.19 ± 0.041 c	< LOD

C – corn by-product samples; con – control samples (non-extruded, non-fermented); Lpl, Lu, Lc, Lpa – fermented with *L. plantarum*-LUHS122, *L. uvarum*-LUHS245, *L. casei*-LUHS210, and *L. paracasei*-LUHS244 strains, respectively; ex – extruded samples; 14, 16, 18 – moisture content of the corn by-product samples; < LOD – lower than the limit of detection (LOD values are given in Supplementary Data 3). Data are represented as means (n = 3) ± SE. a–e – mean values within a column denoted with different letters are significantly different ($p \leq 0.05$).
