# Supplementary material for: Combined Thermomechanical–Biological Treatment for Corn By-Product Valorization into Added-Value Food (Feed) Material
Source: Plants (Basel). 2022 Nov 14;11(22):3080. doi: 10.3390/plants11223080 (PMC9696026; doi:10.3390/plants11223080)
Supplement: Supplementary file 1 [file plants-11-03080-s001.zip › Supplementary Data S3. Table S2. LOD values.pdf]

SUPPLEMENTARY DATA

Table S2. Limit of detection values (LOD) for analysed fatty acids.

Fatty acid	LOD, μg/mL	Fatty acid	LOD, μg/mL
C4:0	6.76	C17:2	2.14
C6:0	4.13	C18:0	4.24
C8:0	3.29	C18:1 tr.	2.16
C10:0	2.88	C18:1	3.90
C10:1	2.65	C18:2 tr.	1.88
C10:2	2.73	C18:2	1.87
C11:0	2.46	C20:0	4.01
C11:1	2.87	C18:3	2.47
C11:2	2.21	C20:1	2.30
C12:0	2.87	C18:3	2.17
C12:1	2.53	C18:4	2.11
C12:2	2.42	C21:0	2.07
C13:0	1.46	C20:2	2.10
C13:1	1.15	C22:0	4.40
C13:2	1.43	C20:3	2.28
C14:0	2.96	C22:1	2.40
C14:1	1.65	C20:3	1.74
C14:2	1.55	C23:0	3.45
C15:0	2.11	C20:4	6.52
C15:1	1.94	C22:2	2.40
C15:2	1.84	C24:0	4.47
C16:0	4.93	C20:5	1.68
C16:1	2.25	C24:1	2.51
C16:2	2.10	C22:5	2.84
C17:0	2.13	C22:6	3.84
C17:1	2.15		
